# Effectiveness of introducing pulse oximetry and clinical decision support algorithms for the management of sick children in primary care in India and Tanzania on hospitalisation and mortality: the TIMCI pragmatic cluster randomised controlled trial

**DOI:** 10.1016/j.eclinm.2025.103306

**Published:** 2025-07-03

**Authors:** Fenella Beynon, Grace Mhalu, Divas Kumar, Silvia Cicconi, Hélène Langet, Gillian A. Levine, Girdhar Agarwal, Charles Festo, Tracy R. Glass, Anmol Jacob, Gaurav Kumar, Samwel Lwambura, Lena Matata, Abdallah Mkopi, Robert Moshiro, Fabian Schaer, Vineela Bandi, Leah F. Bohle, Mira Emmanuel-Fabula, Megan Horlacher, Susan Horton, John Maiba, Suzan Makawia, Naomi Masanja, Deusdedit Mjungu, Ibrahim E. Mtebene, Olgah Odek, Vânia Oliveira, Elena Pantjushenko, Elisabeth Reus, Michael Ruffo, Kovid Sharma, Helen Storey, Mansi Tyagi, Valérie D'Acremont, Honorati Masanja, Shally Awasthi, Kaspar Wyss, the TIMCI Collaborator Group, Agarwal Girdhar, Arimi Peter, Awasthi Shally, Ba Maymouna, Bandi Vineela, Beynon Fenella, Leah F. Bohle, Bulo Method, Cicconi Silvia, Cissé Magib, Clemence Zach, Cleveley Lisa, Cummings Ray, D'Acremont Valérie, Emmanuel-Fabula Mira, Faivre Vincent, Faye Mouhamadou Mansour, Faye Papa Moctar, Festo Charles, Tracy R. Glass, Gupta Kanishka, Harner-Jay Claudia, Horlacher (Shawcross) Megan, Horton Susan, Jacob Anmol, Keitel Kristina, Keith Bonnie, Kosgei Rose, Kumar Divas, Kumar Gaurav, Lalwani Tanya, Langet Hélène, Levine Gillian, Lwambura Samwel, Machoki James, Maiba John, Makawia Suzan, Mansi Tyagi, Martin Gregory, Masanja Honorati, Masanja Irene, Masanja Naomi, Matata Lena, Mhalu Grace, Miheso Andolo, Mjungu Deusdedit, Mkopi Abdallah, Moshiro Robert, Mtebene Ibrahim, Mugo Mercy, Ndiaye Ousmane, Ngari Kevin, Ngutu Mariah, Njiri Francis, Norris Martin, Odek Olgah, Oliveira Vânia, Onah Michael, Orschulko Anja, Oviedo Dickens, Pantjushenko Elena, Quintanar Solares Manjari, Rajaratnam Julie, Rastogi Tuhina, Reus Elisabeth, Ruffo Mike, Schär Fabian, Schaufelberger Sylvain, Sharma Kovid, Shauri Janet, Smith Lisa, Sougou Ndèye Marème, Storey Helen, Tan Rainer, Thabard Julian, Thiongane Aliou, Tine Jean Augustin Diégane, Vonlanthen Alan, Wyss Kaspar

**Affiliations:** a Swiss Tropical and Public Health Institute, Allschwil, Switzerland; b University of Basel, Basel, Switzerland; c Ifakara Health Institute, Dar es Salaam, United Republic of Tanzania; d Department of Pediatrics, King George Medical University, Lucknow, Uttar Pradesh, India; e Department of Pediatrics and Child Health, Muhimbili University Health and Allied Sciences, Dar es Salaam, United Republic of Tanzania; f PATH; g School of Public Health and Health Systems, University of Waterloo, Waterloo, Canada; h Centre for Primary Care and Public Health (Unisanté), University of Lausanne, Switzerland

**Keywords:** Pulse oximetry, Hypoxaemia, Clinical decision support, Integrated management of childhood illness, Primary care, Child health, Randomised controlled trial

## Abstract

**Background:**

Pulse oximetry can support better detection of hypoxaemia, an important mortality predictor, and digital clinical decision support algorithms (CDSAs) can strengthen adherence to Integrated Management of Childhood Illness (IMCI) guidelines. This study sought to address evidence gaps on the impact of providing these tools to primary care healthcare providers on under-five hospitalisations and mortality.

**Methods:**

A pragmatic, parallel group, superiority, cluster randomised controlled trial (RCT) conducted in 172 primary care facilities in India and Tanzania (106 and 66 facilities, respectively). Facilities were randomly allocated (1:1) in India to pulse oximetry (PO) or control and (1:1:1) in Tanzania to PO + CDSA, PO, or control, stratified by facility type and location (India: district; Tanzania: urban/rural). Sick children aged 0–59 months attending study facilities were eligible. Pulse oximeters and CDSAs were given to healthcare providers, along with training and guidance, supportive supervision, monitoring, community engagement, and operational support. Providers were advised to use pulse oximetry for all sick children in India, and in Tanzania for all 1–59 days, and for those 2–59 months with cough, difficulty breathing, or a moderate to severe illness. Urgent referral was recommended for SpO_2_ <90%. Trained research assistants collected data from caregivers and facility records on Day 0, with a follow-up phone call or visit on Day 7 and 28. Two primary outcomes, based on caregiver report, were assessed centrally: 1) rates of ‘severe complication’ (death, delayed hospitalisation (≥24 h from the Day 0 consultation) or hospitalisation without Day 0 referral) by Day 7; and 2) rates of hospitalisation within 24 h of the Day 0 consultation, with referral. Intention-to-treat analyses were performed on combined and individual country data, stratified by age (1–59 days, 2–59 months). Primary outcomes were assessed using generalised estimating equations for logistic regression, with facilities as clusters. Results were estimated in terms of odds ratios and risk differences (RDs), adjusted where computable. The trial is registered with clinicaltrials.gov (NCT04910750).

**Findings:**

A total of 157,677 sick children (1–59 days: 3188 control, 4012 PO, 2386 PO + CDSA; 2–59 months: 54,318 control, 56,968 PO, 36,805 PO + CDSA) were enrolled from 28 March, 2022 to 31 March, 2023 in Tanzania and from 20 June, 2022 to 21 April, 2023 in India. Severe complications were rare in the control arm, with 16 (0·5%) events in 1–59 days, 77 (0·1%) in 2–59 months. No significant difference was observed in 1–59 days in the PO arm, with 27 events (0·7%, RD 0·2% [−0·2%; 0·5%]), but a slight increase was noted in 2–59 months, with 143 events (0·3%, adjusted RD 0·1% [0·0%; 0·2%]). No statistically significant differences were observed in the CDSA + PO arm, with 21 events (0·9%, RD 0·5% [−0·1%, 1·0%]) in 1–59 days, 128 (0·3%, adjusted RD 0·1% [−0·0%, 0·3%]) in 2–59 months. Day 0 hospitalisations with referral were very rare in the control arm, with 0 events (0·0%) in 1–59 days, 12 (0·0%) in 2–59 months. Arm comparisons were either not computable or not statistically significant, within the PO arm: 9 events (0·2%) in 1–59 days, 22 (0·0%, RD 0·0% [−0·0%, 0·1%]) in 2–59 months; in the CDSA + PO arm: 6 events (0·3%) in 1–59 days, 32 (0·1%, RD 0·0% [−0·0%, 0·1%]) in 2–59 months.

**Interpretation:**

When implemented in routine health systems at primary care level in India and Tanzania, contrary to expectations, pulse oximetry and CDSAs were not found to increase rates of hospitalisation within 24 h of primary care referral, nor to decrease deaths, or delayed or un-referred hospitalisations. Wider health system challenges, including referral barriers, inequitable oxygen access and hospital care quality must be addressed if the potential of these tools in delivering child outcome benefits is to be realised.

**Funding:**

Unitaid grant n°2019-35-TIMCI: Tools for Integrated Management of Childhood Illness.

**Research in context**

**Evidence before this study**

Integrated Management of Childhood Illness (IMCI) guidelines are widely used in resource-constrained settings but often miss hypoxaemia, a key predictor of mortality, and are inconsistently followed. Pulse oximetry and digital clinical decision support algorithms (CDSAs) are two strategies designed to improve the identification and management of severe illness in primary care. Both have shown promise in strengthening assessment and referral decisions. However, most evidence comes from observational studies, and findings on patient outcomes such as hospitalisation and mortality remain limited and inconclusive.

**Added value of this study**

To our knowledge, this is the first multi-country pragmatic cluster RCT powered to assess the impact of primary care introduction of pulse oximetry, with or without clinical decision support, on hospitalisations and death of children under five. Despite small increases in urgent referrals, we did not find a reduction in severe complications, nor a significant increase in hospitalisations within 24 h and as a result of referral.

**Implications of all the available evidence**

There is strong evidence that hypoxaemia is associated with mortality and cannot reliably be detected by IMCI clinical signs alone, and that severe illness often goes undetected due to low IMCI adherence. A number of studies have demonstrated the relevance of pulse oximetry in supporting primary healthcare providers to identify severe hypoxaemia, and of CDSAs in strengthening guideline adherence. The finding of this study that hospitalisation rates and mortality improvements are not realised with the introduction of these devices indicates the need to prioritise accompanying broader health systems strengthening efforts—to improve access to and overall quality of primary and hospital care and strengthen oxygen systems.

## Introduction

Children under five years of age in low-and middle-income countries face an unacceptable burden of preventable morbidity and mortality.^1^ Hypoxaemia—a low blood oxygen level (or oxygen saturation, SpO_2_)—is an important predictor of mortality among sick children under-five,^2^, ^3^, ^4^ but is not reliably detected by assessment of clinical signs included in Integrated Management of Childhood Illness (IMCI) guidelines.^5^, ^6^, ^7^, ^8^, ^9^

Pulse oximeters provide a quick and non-invasive method of evaluating hypoxaemia, with several studies demonstrating acceptability and feasibility in primary care in resource-constrained settings.^7^^,^^10^^,^^11^ Evidence also suggests that pulse oximetry can improve severe illness detection at the primary care level, compared with using IMCI clinical signs alone.^6^, ^7^, ^8^, ^9^^,^^12^

Yet evidence on the impact of pulse oximetry on outcomes beyond the primary care visit is very limited.^13^ Modelling suggests that introducing pulse oximetry could prevent 150,000 child deaths annually in the 15 highest pneumonia burden countries.^14^ However, the model's assumptions relied on limited data from hospitals, where pulse oximetry was combined with support to oxygen systems, rather than primary care. Beyond this study, most evidence is observational, with few studies evaluating caregiver adherence to referral advice, hospitalisations, and mortality with pulse oximetry introduction.^6^^,^^8^ These gaps have been highlighted by WHO as a key barrier to scale-up, and the recent Lancet Commission on Medical Oxygen Security underscores the need for pragmatic research on pulse oximetry introduction in primary care, particularly on referral and clinical outcomes.^13^^,^^15^

In addition, evidence from hospital settings highlights the importance of hypoxaemia among sick young infants (1–59 days) and children with non-pneumonia diagnoses. However, data are lacking on presenting clinical syndromes to determine whether the current IMCI recommendation^16^ for pulse oximetry should extend beyond children aged 2–59 months with cough or difficulty breathing.^6^^,^^17^, ^18^, ^19^, ^20^

The challenge that IMCI does not reliably detect hypoxaemia is compounded by inconsistent IMCI adherence by healthcare providers, resulting in both under-detection of severe illness and inappropriate treatment.^21^^,^^22^ Digital clinical decision support algorithms (CDSAs), which provide step-by-step, patient-tailored guidance, are recommended by WHO to strengthen guideline adherence.^23^ Several IMCI-based CDSAs have been implemented, with resulting improvements in clinical assessment completeness, classification accuracy, treatment appropriateness, and reduced antibiotic prescription. However, a recent systematic review highlighted the heterogeneity of findings and paucity of evidence on outcomes beyond the primary care visit.^24^^,^^25^

The Tools for Integrated Management of Childhood Illness (TIMCI) project aimed to support healthcare providers identify and manage severe illness among children under-five, by introducing pulse oximetry and CDSAs to primary care facilities in India, Kenya, Senegal and Tanzania. Accompanying the ‘real-world’ implementation, a large-scale, multi-country, mixed method evaluation was conducted to address evidence gaps and inform national and international decision-making on scale-up.^26^ This article focuses on whether pulse oximetry, with or without CDSAs, results in improved clinical outcomes of sick children under-five attending primary care, based on findings from the pragmatic cluster RCT in India and Tanzania; a parallel article presents findings from the quasi-experimental pre-post study in Kenya and Senegal.^27^

## Methods

### Study design

A pragmatic, parallel group, superiority, cluster randomised controlled trial (RCT) compared clinical care and outcomes of children attending government primary care facilities (clusters) randomly allocated to 1) pulse oximetry combined with a digital clinical decision support algorithm (PO + CDSA), 2) pulse oximetry alone (PO) or 3) control, defined as usual care. Prior to the RCT, a 3-month pilot informed intervention adaptations, including a decision not to proceed with the PO + CDSA arm in India, based on a need for substantial CDSA adaptation and further piloting before effectiveness evaluation. Further details on the study design are described elsewhere.^26^ Ethical approval was obtained from the King George's Medical University Internal Ethics Committee (ECR/262/Inst/UP/2013/RR-16), the Indian Council of Medical Research (2020–9753), the Ifakara Health Institute Institutional Review Board Ref IHI/IRB/AMM/01–2023, the Tanzania National Institute for Medical Research (NIMR/HQ/R.8c/Vol. I/2265), and the WHO Ethics Review Committee (ERC.0003405, v2·4, 2 February 2023).

### Setting and participants

The RCT was conducted in government primary health centres (PHCs) and community health centres (CHCs) in Uttar Pradesh (Deoria, Sitapur, Unnao) and dispensaries and health centres in Tanzania (Kaliua, Sengerema, Tanga), as shown in Fig. 2A. Facilities providing curative primary care services for under-fives, with access to oxygen (on-site or at the designated referral facility) and electricity (continuous or intermittent) were eligible. Facilities were excluded if inaccessible to the study team, saw fewer than 20 sick children per month, already systematically used pulse oximetry within IMCI, or had another major child health intervention planned during the study.

**Fig. 1:**
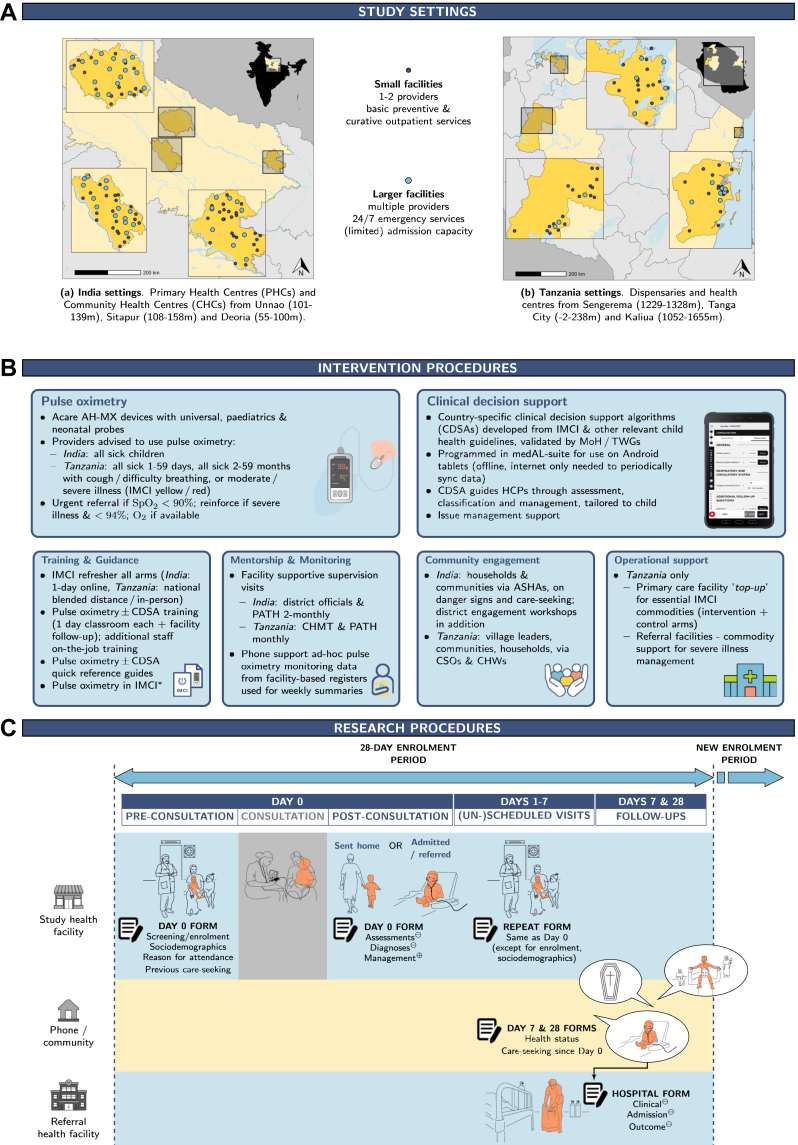
Overview of the study settings (Panel A), implementation (Panel B), and research procedures (Panel C). Panel A: Spatial distribution of facilities by type, with the average altitude of each area provided in metres (m). Panel B: The asterisk (∗) refers to IMCI chart booklet updated to include pulse oximetry according to country specific criteria. ASHAs, Accredited Social Health Activist; CHMT, Council Health Management Team; CHWs, Community Health Workers; CSOs, Civil Society Organisations; MoH, Ministry of Health; TWGs, Technical Working Groups. Panel C: Data sources are indicated as follows: ⊝ = facility records only; ⊕ = both caregivers and facility records; otherwise, from caregivers only.

**Fig. 2:**
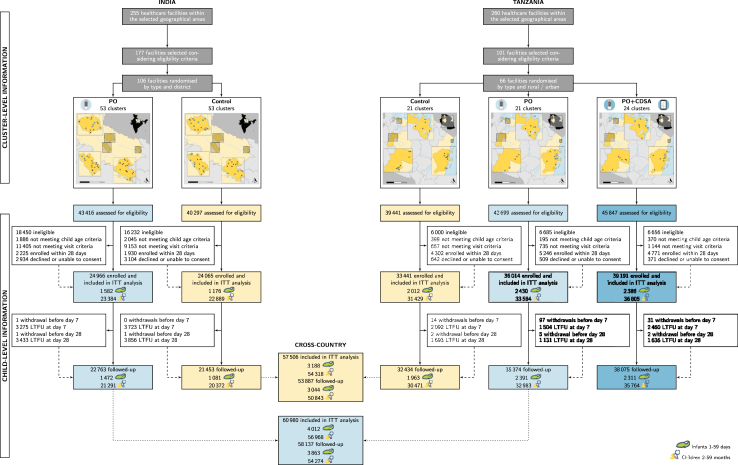
Study flowchart by intervention period, cross- and by country. Numbers are disaggregated by age groups (1–59 days, 2–59 months). Yellow boxes correspond to the control arm; light blue boxes to the PO arm; blue boxes to the PO + CDSA arm. ITT, intention-to-treat; LTFU, lost-to-follow-up.

Children aged 0–59 months were eligible if they attended a study facility for an illness or were reported unwell during a routine visit. Children on their first day of life, attending for trauma only, already an inpatient, or enrolled within the preceding 28 days, were excluded.

### Randomisation and masking

Clusters were randomly allocated (1:1) in India to PO or control and (1:1:1) in Tanzania to PO + CDSA, PO or control from eligible facilities by an independent statistician, stratified by facility type (India: PHC/CHC; Tanzania: dispensary/health centre) and location (Tanzania: urban/rural; India: district, given all facilities were classified as rural). Unallocated eligible facilities were retained for later allocation if needed. Given the cluster design, concealment only occurred at facility allocation, conducted centrally and distributed to study sites.

### Procedures

The TIMCI intervention package (Fig. 2B) included pulse oximeters, with or without CDSA, along with training and guidance, supportive supervision, routine monitoring, community engagement, and operational support. The ‘global’ package was developed collaboratively, based on evidence, institutional experience, and stakeholder consultation. Country-specific adaptations were conducted with Ministries of Health (MoHs) and other stakeholders.

Acare AH-MX pulse oximeters were provided with universal, paediatric and neonatal probes, with guidance (in an adapted chart booklet and the CDSA) to use them in all consultations with sick children 0–59 months in India, and in Tanzania for all 1–59 days and for 2–59 months with cough or difficulty breathing, or a moderate to severe illness (yellow/red IMCI classification). Urgent referral was recommended for children with SpO_2_ <90%, in line with national IMCI. In Tanzania, the CDSA was based on national IMCI and other relevant national and global child health guidelines.^28^ In addition to intervention arm pulse oximetry ± CDSA training, IMCI refresher training was provided in all arms to reduce potential bias from a training effect.

Children attending study facilities were screened prior to consultations, and enrolled if eligible and following caregiver (parent or other guardian) written informed consent, or a thumbprint and impartial witness signature if illiterate. Critically unwell children were only enrolled if possible following stabilization. Data were collected by trained research assistants. On Day 0 (D0), this included information on care-seeking, clinical presentation, measurements, diagnoses, treatments and other management including referral, from brief caregiver interviews before and after consultation, and from facility records (Fig. 2C). Caregiver-reported recovery and subsequent care-seeking were collected on Day 7 (D7) and 28 (D28) by phone, with community follow-up for caregivers unreachable by phone. Data were also collected at study facilities for any child attending within the follow-up period, though priority was given to new patient recruitment—(un-)scheduled follow-up was therefore opportunistic. Basic hospital attendance and admission data were collected if the child was reported to have attended hospital.

### Outcomes

Two primary outcomes were assessed centrally: 1) rates of ‘severe complication’ (death, delayed hospitalisation (>24 h from the D0 consultation) or hospitalisation without D0 referral) by D7; and 2) rates of hospitalisation ≤24 h of the D0 consultation, with referral. We hypothesised that the intervention would result in reduced ‘severe complications’ and increased hospitalisations ≤24 h with referral, due to better detection and referral of severely ill children. Though reliant on factors beyond the influence of the intervention, particularly access to quality hospital care, these outcomes were selected based on stakeholder priorities of evidence about impact of the interventions on outcomes beyond primary care, rather than on hypoxaemia detection and healthcare provider decision-making.

Secondary outcomes, detailed elsewhere (NCT04910750), included: hypoxaemia prevalence (SpO_2_ <90%, 90–91%, and 92–93%); severe complications according to hypoxaemia status; urgent referrals; referral completion; oxygen administration; outcomes related to antibiotic prescription and malaria testing and treatment; (un-)scheduled primary care follow-up; and caregiver-reported recovery at D7.

Pulse oximetry uptake was calculated as the proportion of children with documented SpO_2_ among those for whom it was indicated, i.e. all 1–59 days (both countries), all 2–59 months (India) and 2–59 months with cough/difficulty breathing or moderate/severe illness based on caregiver report or recorded diagnoses (Tanzania). CDSA uptake was estimated as the number of CDSA consultations compared to the number of enrolled children.

### Statistical analysis

#### Sample size

Sample sizes were calculated separately for India and Tanzania considering anticipated facility enrolment rates over a planned 12 month recruitment period, for each arm compared to control, to detect a ≥30% decrease in severe complications (from 1·1%^29^) and ≥30% increase in hospitalisations ≤24 h of the D0 consultation, with referral (from facility estimates of 1·5%), with 80% power, 0·05 Type I error, and intra-cluster correlation coefficient of 0·001,^30^ without adjustment for drop-out given that substantial efforts were made to minimize loss-to-follow-up. In Tanzania, 22 clusters per arm, recruiting an average of 1680 children (total 110,880), were required; and in India, 40 clusters per arm, recruiting an average of 510 children (total 40,800).

#### Analysis

Intention-to-treat (ITT) analyses were performed on combined and individual country data. Baseline characteristics and outcomes were described by study arm, stratified by age (1–59 days, 2–59 months), with summary statistics. Primary outcomes were assessed using generalised estimating equations for logistic regression (gee R package), with facilities as clusters. Models were adjusted for individual-level baseline variables found to be randomly imbalanced across arms. Modelling for sensitivity analyses and secondary outcomes were performed in a similar way, when numbers allowed. Binary outcomes were reported with odds ratios (ORs) andrisk differences (RDs) derived using a marginal standardization approach (emmeans R package) with 95% confidence intervals (95% CIs), continuous outcomes with adjusted mean differences and 95% CIs. Primary outcomes were evaluated with a hierarchical fallback procedure using a weighted Bonferroni calculation, recycling unspent significant levels to test pre-specified subsequent hypotheses.^31^, ^32^, ^33^ Sensitivity analyses were performed: using only the first disease episode of each child during the study period; in case of substantial missing data (best/worst case scenario, complete cases, multiple imputation with chained equations); for different definitions of delayed hospitalisations; and for urgency of referral. All analyses were performed using R4·1·2.

#### Monitoring

The trial was independently monitored in accordance with Good Clinical Practice, and overseen by an independent Data Monitoring Committee, according to the published Charter (NCT04910750).

### Role of funding source

The funder had no role in study design, data collection, data analysis, data interpretation, or writing of the manuscript.

## Results

In total, 74 facilities were randomly assigned (53 in India; 21 in Tanzania) to the control arm, 74 (53 in India; 21 in Tanzania) to the PO arm, and 24 to the PO + CDSA arm in Tanzania. Enrolment occurred from March 28, 2022 to March 31, 2023 in Tanzania and from June 20, 2022 to April 21, 2023 in India.

### Population

The flowchart for the trial is detailed in Fig. 1. A total of 157,677 children were enrolled: 57,506 (24,065 in India; 33,441 in Tanzania) in the control arm, 60,980 (24,966 in India; 36,014 in Tanzania) in the PO arm, and 39,191 in the PO + CDSA arm. Recruitment details by facility characteristics can be found in Supplement S1.

The demographic and clinical characteristics of the trial population are summarized in Table 1. Infants 1–59 days accounted for 5·6% of all children in India, 6·3% in Tanzania. Overall, cross-country characteristics were balanced across arms, though variations in care-seeking behaviours and clinical presentation emerged at the country level. In Tanzania, caregivers reported having sought prior care at health facilities more frequently in the PO (7·9%) and PO + CDSA (8·3%) arms than in the control arm (4·5%). In India, caregivers reported severe symptoms more frequently in the control than in the PO arm (4·1% vs. 1·4% children with a least one danger sign; 1·2% vs. 0·7% with difficulty breathing). In addition, care-seeking characteristics generally differed between India and Tanzania.

**Table 1:** Demographic and clinical characteristics of children 1 day to 59 months enrolled in the TIMCI study by intervention arm, cross- and by country.






Yellow columns correspond to the control arm; light blue columns to the PO arm; blue columns to the PO + CDSA arm.

^1^These questions allowed for multiple answers, therefore options are not mutually exclusive.

^2^These are only evaluated for children presenting with the referenced sign/symptom.

Primary outcomes for ITT analyses were available for 93·7% of children (89·1% in India; 97·0% in Tanzania) in the control arm, 95·3% (91·2% in India; 98·2% in Tanzania) in the PO arm, and 97·2% in the PO + CDSA arm. Characteristics of children lost to follow-up were similar to those with follow-ups (Supplement S2). Multivariate models were adjusted for districts, facility type and prior care or treatment.

### Intervention uptake

In the PO arm, 59·9% of 1–59 days and 71·0% of 2–59 months with pulse oximetry indication had SpO_2_ values documented in facility records. A marked contrast was observed between countries, with pulse oximetry uptake in India (94·4% in 1–59 days; 94·5% in 2–59 months) about two-fold higher than in Tanzania (37·4% in 1–59 days; 48·0% in 2–59 months with pulse oximetry indication. While CDSA uptake was estimated to be relatively high in the PO + CDSA arm (75·0%), pulse oximetry uptake, as documented in facility records, trended slightly lower in this arm than in the PO arm in Tanzania for both age groups (33·6% in 1–59 days; 45·7% in 2–59 months with pulse oximetry indication). Of note, there was some limited use of pulse oximetry in the control arm in India (3·4% in 1–59 days and 2·4% in 2–59 months).

### Hypoxemia prevalence

A small proportion of children had documented severe hypoxaemia (SpO_2_ <90%), with prevalence 1·3 to 2·3 times higher in 1–59 days (0·5% overall; 0·7% in India; 0·4% in the PO arm and 0·5% in the PO + CDSA arm in Tanzania) compared to 2–59 months (0·3%, consistent across countries and arms). In India, the prevalence of SpO_2_ 90–91% was comparable to that of SpO_2_ <90%, while SpO_2_ 92–93% was twice as frequent. In Tanzania, these rates were three and ten times higher that of SpO_2_ <90%, with a slightly higher prevalence in the PO + CDSA than in the PO arm (Table 2).

**Table 2:** Pulse oximetry uptake and hypoxaemia.

Characteristics	1–59 days				2–59 months			
	PO			PO + CDSA	PO			PO + CDSA
	Cross-country (n = 4012)	India (n = 1582)	Tanzania (n = 2430)	Tanzania (n = 2386)	Cross-country (n = 56,968)	India (n = 23,384)	Tanzania (n = 33,584)	Tanzania (n = 36,805)
Eligible for pulse oximetry, % (n)	4012 (100·0%)	1582 (100·0%)	2430 (100·0%)	2386 (100·0%)	47,223 (82·9%)	23,384 (100·0%)	23,839 (71·0%)	27,437 (74·5%)
Pulse oximetry uptake, % (n/N)^a^	2403 (59·9%)	1493 (94·4%)	910 (37·4%)	802 (33·6%)	33,545 (71·0%)^f^	22,098 (94·5%)	11,447 (48·0%)^f^	12,535 (45·7%)^f^
**Hypoxaemia, % (n)**								
<90%	20 (0·5%)	11 (0·7%)	9 (0·4%)	12 (0·5%)	170 (0·3%)	79 (0·3%)	91 (0·3%)	93 (0·3%)
90%–<92%	57 (1·4%)	16 (1·0%)	41 (1·7%)	39 (1·6%)	397 (0·7%)	56 (0·2%)	341 (1·0%)	405 (1·1%)
92%–<94%	97 (2·4%)	23 (1·5%)	74 (3·0%)	81 (3·4%)	798 (1·4%)	123 (0·5%)	675 (2·0%)	1026 (2·8%)
**Severe complications by SpO_2_ group, % (n/N)**^b^								
Normoxaemic (≥94%)	23 (1·0%)	17 (1·2%)	6 (0·8%)	8 (1·2%)	99 (0·3%)	51 (0·2%)	48 (0·4%)	77 (0·6%)
<90%	3 (15·0%)	2 (18·2%)	1 (11·1%)	0 (0·0%)	9 (5·3%)	7 (8·9%)	2 (2·2%)	7 (7·5%)
90%–<92%	4 (7·0%)	3 (18·8%)	1 (2·4%)	1 (2·6%)	8 (2·0%)	4 (7·1%)	4 (1·2%)	5 (1·2%)
92%–<94%	1 (1·0%)	1 (4·3%)	0 (0·0%)	0 (0·0%)	8 (1·0%)	5 (4·1%)	3 (0·4%)	9 (0·9%)
<40% (spurious)	0 (0·0%)		0 (0·0%)	0 (0·0%)	0 (0·0%)	0 (0·0%)	0 (0·0%)	0 (0·0%)
Unknown SpO_2_^c^	15/1617 (0·9%)	2/91 (2·2%)	13/1526 (0·9%)	25/1590 (1·6%)	99/20,456 (0·5%)	2/1312 (0·2%)	97/19,144 (0·5%)	108/21,776 (0·5%)
**Cascade of care in children with SpO_2_ <90%, % (n)**								
Urgent referrals^d^	11/20 (55·0%)	6/11 (54·5%)	5/9 (55·6%)	8/12 (66·7%)	30/170 (17·6%)	14/79 (17·7%)	16/91 (17·6%)	46/93 (49·5%)
Hospital attendances among referred^e^	6/11 (54·5%)	3/6 (50·0%)	3/5 (60·0%)	0/8 (0·0%)	9/30 (30·0%)	6/14 (42·9%)	3/16 (18·8%)	9/46 (19·6%)
Hospital admissions among referred^e^	4/11 (36·4%)	2/6 (33·3%)	2/5 (40·0%)	0/8 (0·0%)	4/30 (13·3%)	1/14 (7·1%)	3/16 (18·8%)	8/46 (17·4%)
Oxygen administration among referred^e^	0/11 (0·0%)	0/6 (0·0%)	0/5 (0·0%)	0/8 (0·0%)	1/30 (3·3%)	1/14 (7·1%)	0/16 (0·0%)	0/46 (0·0%)

a The denominator of each proportion is the group identified in the row immediately above.

b The denominator of each proportion is the corresponding SpO_2_ group, including those for whom pulse oximetry was performed but not indicated.

c The denominator includes both children who did not receive a pulse oximetry measurement and those whose pulse oximetry measurements were unreadable.

d The denominator of each proportion is the population with SpO2 <90%, i.e. a value prompting an urgent referral recommendation.

e The denominator of each proportion is the population with SpO2 values prompting an urgent referral recommendation who was referred at the D0 consultation.

f Pulse oximetry may still have been used in children 2–59 months who were not eligible for it.

### Primary outcomes

The unexpectedly low number of events limited the feasibility of multivariate modelling and affected model precision for primary outcomes.

**Fig. 3:**
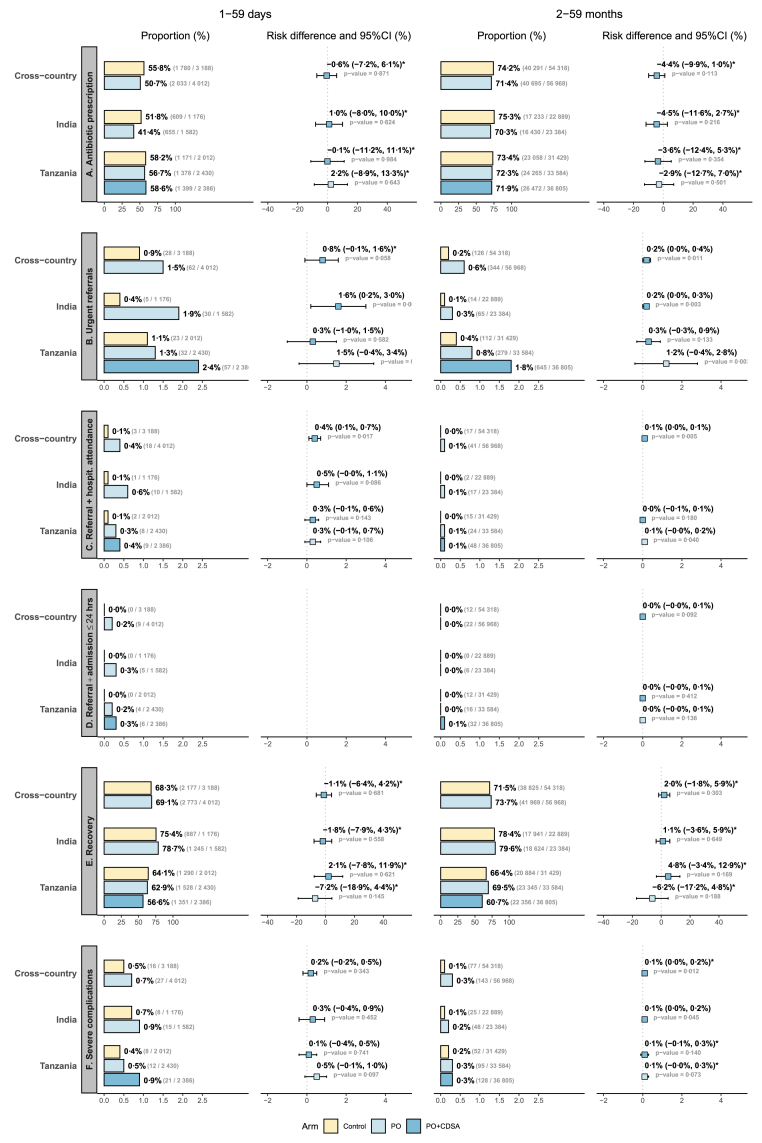
Outcome proportion and forest plot comparison between the control, PO and PO + CDSA arms. Yellow bars correspond to the control arm; light blue bars to the PO arm; and blue bars to the PO + CDSA arm. Light blue forest plots correspond to PO vs. control; blue forest plots correspond to PO + CDSA vs. control. Asterisks indicate RD and 95% CI resulting from adjusted models (unadjusted models otherwise). CI, Confidence Interval.

Fewer children than expected experienced severe complications by D7 in the control arm (Fig. 3F), with rates five times higher in 1–59 days (0·5%; 16 infants) than in 2–59 months (0·1%; 77 children). Secondary hospitalizations contributed more than deaths to severe complications (Supplement S3). Event rates trended consistently higher in intervention arms, which was unexpected. No statistically significant difference was seen in 1–59 days in the PO arm, with 27 (0·7%, RD 0·2% [−0·2%; 0·5%]) severe complications, 15 (0·9%, RD 0·3% [−0·4%; 0·9%]) from India and 12 (0·5%, RD 0·1% [−0·4%; 0·5%]) from Tanzania. However, a slightly significant increase was observed in 2–59 months both cross-country (143 children, 0·3%, adjusted RD 0·1% [0·0%; 0·2%]) and in India (48 children, 0·2%, RD 0·1% [0·0%; 0·2%]), but not in Tanzania (95 children, 0·3%, adjusted RD 0·1% [−0·1%; 0·3%]). No statistically significant differences were observed in the CDSA + PO arm where 21 (0·9%, RD 0·5% [−0·1%; 1·0%]) 1–59 days and 128 (0·3%, adjusted RD 0·1% [−0·0%; 0·3%]) 2–59 months experienced severe complications. Severe complications were more common in children with SpO_2_ <90% and 90–91% than in normoxaemic children (Table 2).

Very few children in the control arm were referred on D0 and hospitalized ≤24 h (Fig. 3D): 0 (0·0%) in 1–59 days and 12 (0·0%)—all from Tanzania (0·0%)—in 2–59 months. Although rates were consistently higher in the intervention arms, the strength of association (ORs, see Supplement S4) and risk differences (RDs) were either not computable or not statistically significant. In the PO arm, 9 (0·2%) 1–59 days—5 (0·3%) from India, 4 (0·2%) from Tanzania—and 22 (0·0%, RD 0·0% [−0·0%; 0·1%]) 2–59 months—6 (0·0%) from India, 16 (0·0%, RD 0·0% [−0·0%; 0·1%]) from Tanzania—were referred and hospitalized ≤24 h. In the CDSA + PO arm, 6 (0·3%) 1–59 days and 32 (0·1%, RD 0·0% [−0·0%; 0·1%]) 2–59 months were referred and hospitalized ≤24 h.

Results remained consistent across the different sensitivity analyses (Supplement S4).

### Care cascade

Few children were referred to a higher level of care in the control arm (Fig. 3B), with referral rates 4·5-fold higher in 1–59 days (0·9%; 28 infants) than in 2–59 months (0·2%; 126 children). Referral rates were consistently higher in the intervention arms with statistically significant differences observed in the PO arm both cross-country and in India for both age groups. No statistically significant differences were observed in Tanzania, although ORs were significant in the PO + CDSA arm (Supplement S5).

Very few children were referred and subsequently reported by caregivers to have attended a higher level of care in the control arm (Fig. 3C): 3 (0·1%) in 1–59 days and 17 (0·0%) in 2–59 months. Completed referral rates were consistently higher in intervention arms, with slightly statistically significant unadjusted differences observed in the PO arm cross-country for both age groups, but not at the individual country level. Unadjusted ORs were also statistically significant in the PO + CDSA arm, but not RDs (Supplement S5).

Among children with SpO_2_ <90%, 55·0% (11/20) of 1–59 days and 17·6% (30/170) of 2–59 months in the PO arm were referred to a higher level of care, with consistent rates across countries (Table 2). Among these referrals, caregivers reported 54·5% hospital attendances and 36·4% admissions in 1–59 days, with similar rates in both countries, and 30·0% attendances and 13·3% admissions in 2–59 months, with higher attendance in India but higher admission rates in Tanzania. Although most hospital records could be found when children were admitted (Supplement S6), hospital-administered oxygen was documented in 3·3% (1/30) of 2–59 months only and none of 1–59 days. In the PO + CDSA arm, referral rates for children with SpO_2_ <90% were higher in 1–59 days (66·7%) than in 2–59 months (49·5%). However, no hospital attendances or admissions were reported in 1–59 days, and hospital attendance and admission rates did not improve in 2–59 months; no records of hospital-administered oxygen were found.

### Other secondary outcomes

A majority of children in the control arm were prescribed antibiotics during the D0 consultation (Fig. 3A), with rates 1·3-fold higher in 2–59 months (74·2%) than in 1–59 days (55·8%), consistent across countries. Prescription rates in intervention arms remained statistically stable but trended lower for all comparisons. Recovery rates reported by caregivers at D7 also remained statistically stable compared to the control arm (68·3% in 1–59 days; 71·5% in 2–59 months), although contrasting variations were observed cross- and by country, across intervention arms and age groups (Fig. 3E). Further details on the remaining secondary outcomes, which require careful interpretation, are provided in Supplement S5.

## Discussion

The TIMCI pragmatic cluster RCT, enrolling 157,677 sick children across 172 primary care facilities in India and Tanzania, is the largest study to date evaluating pulse oximetry in primary care, with or without CDSAs, and one of the few to evaluate outcomes beyond the primary care visit. The key finding was that the anticipated impact on hospitalisations and mortality was not observed: there was no significant increase in hospitalisation rates within 24 h of primary care consultations, with referral; and, though events were very rare, we observed slightly higher severe complication (death, or delayed or un-referred hospitalisations) rates for children 2–59 months. Importantly, this was in the context of high intervention uptake in India and moderate uptake in Tanzania, relatively low prevalence of hypoxaemia (SpO_2_ <90%), and higher urgent referral rates by primary care healthcare providers in intervention arms suggestive of improved severe disease detection. Collectively these findings point to major barriers in access to quality hospital care for children with severe illness, and the critical importance of broader health systems strengthening.

Previous studies indicate high acceptability and uptake of pulse oximetry in primary care, though many have only included children 2–59 months with pneumonia.^7^^,^^10^^,^^11^^,^^34^ In this study, pulse oximetry uptake was close to 95% for both age groups in India, where it was recommended for all sick children. In contrast, despite being recommended for all young infants in Tanzania, only one third had a documented measurement, consistent with known measurement challenges in this group.^11^ Although uptake was only 50% among children 2–59 months with cough, difficulty breathing, or moderate to severe illness in Tanzania, the estimate is uncertain as the denominator was based on caregiver report and recorded diagnoses rather than consultation observations. Marked variation in uptake may be due to contextual or implementation differences, including that India's ‘all sick children’ criteria may have been simpler to implement, though differences may also result from variations in SpO_2_ documentation practice. These possibilities will be explored with the TIMCI mixed-method studies; however, it will be important for policymakers to consider hypoxaemia prevalence, time burden and potential for task-shifting when determining recommendations for different settings.

Healthcare providers equipped with pulse oximetry documented severe hypoxaemia in 0·5% sick young infant consultations and 0·3% sick 2–59 months child consultations. Higher prevalence was expected in Tanzania than India, given the higher burden of morbidity and mortality,^1^ and higher average altitude of included facilities^26^; however this was not observed, possibly due to uptake, documentation, or true epidemiological differences. Hypoxaemia prevalence was low compared to previous estimates, though many are from sub-groups with more severe presentations, such as children with WHO-defined pneumonia (8% non-severe, 41% severe),^35^ referred from primary care (9%),^18^ or at hospital level (12%).^17^ The few studies evaluating prevalence in both respiratory and non-respiratory illness at primary care had similarly low findings: 0·6% among 3000 children in Malawi^19^; 1·3%, among 1575 children in Uganda^6^; and 1·4% among 1663 children in Papua New Guinea.^36^ Variation across countries and with this study may result from differences in uptake, epidemiology (including due to vaccine coverage), health-seeking behaviour, and altitude. Importantly, echoing other studies,^6^^,^^17^^,^^35^ we found higher prevalence among young infants, for whom pulse oximetry is not currently recommended within IMCI.

Pulse oximetry and the CDSA were associated with 2–4-fold higher urgent referral rates, relative to control. This confirm findings from other studies that these tools can help healthcare providers identify children with severe disease.^4^^,^^7^, ^8^, ^9^^,^^12^^,^^37^ Yet urgent referrals for children with hypoxaemia were low, with only 55% of young infants and 18% children 2–59 months referred in the PO arm and 67% and 50% in the PO + CDSA arm in Tanzania, respectively. Among children with and without hypoxaemia, referral completion and hospitalisation within 24 h were very low. Findings from other studies on referral and hospitalisation are mixed: in Ethiopia, providers with pulse oximetry referred 78% of children with severe pneumonia, of whom 53% completed referral^8^; in India, 69% of hypoxaemic children were referred, with 76% referral completion^38^; in Uganda, under half of hypoxaemic children were referred despite free transport provision, with non-referral reasons including provider confidence in ability to manage locally, and caregiver referral refusal or concern about cost^6^; in Malawi, only 46% of children with severe pneumonia were referred, but referrals were higher for children with both hypoxaemia and other severe clinical signs (84%) than with clinical signs alone (42%) or hypoxaemia alone (27%)^7^; and in Bangladesh, 81% of caregivers of children with severe pneumonia declined referral, despite provision of logistical support.^9^ As with other studies, our findings reflect major contextual challenges to referral, from high costs and logistical barriers of hospital attendance and admission, to gender and interpersonal dynamics influencing maternal decision-making, and perceived or actual poor quality of hospital care.^6^^,^^39^^,^^40^

These contextual challenges, and the complex nature of health systems interventions, including variations in implementation fidelity, likely contributed to the lack of reduction in severe complications—a composite outcome of death, or delayed or un-referred hospitalisation. Pulse oximetry at hospitals, particularly combined with oxygen systems, is associated with reduced mortality.^4^^,^^12^ To our knowledge, only one interventional study has explored similar outcomes in primary care, finding improvement in morbidity and mortality with pulse oximetry introduction, but in the context of facility admission capacity and solar-powered oxygen systems.^41^ In contrast, this study used pulse oximetry as a referral tool. Given the strong association of hypoxaemia with mortality,^2^, ^3^, ^4^^,^^37^ and the higher rates of severe complications among hypoxaemic children in this study, future implementation must embed primary care pulse oximetry in wider efforts to strengthen access to quality hospital care, and strengthen capacity for severe illness management, including oxygen, when referral is not feasible. As called for in the recent Lancet Global Health Commission on Medical Oxygen Security, further implementation research is needed to understand the role of contextual factors on adoption, sustainability, and clinical impact of primary care pulse oximetry introduction.^13^

Beyond the lack of improvement, we observed slightly but significantly higher rates of severe complications among children 2–59 months in intervention arms, mainly due to hospitalisation without referral. We identified three potential mechanisms to attribute this to a true effect, along with potential sources of bias. Firstly, healthcare providers may have better identified severe disease and prescribed pre-referral (single dose) treatment which, without hospitalisation, may have resulted in incomplete treatment; conversely, lower severe disease detection and full-course oral treatment could have resulted in better outcomes. This is supported by the higher referral rates in intervention arms, and other studies,^42^^,^^43^ and highlights the importance of close follow-up of children given pre-referral treatment for severe disease. Secondly, device introduction may have distorted care provision—during the consultation (if device use prioritised over other assessments) or at the facility level (introducing delays). Based on other studies and ongoing analyses of TIMCI mixed-method studies, this hypothesis seems unlikely.^7^^,^^8^^,^^12^ Thirdly, though counter to training, healthcare providers may have been falsely reassured by normal SpO_2_ values for children with IMCI severe signs, aligning with findings that children with severe signs have higher referral rates if associated with hypoxaemia.^7^

This study has several limitations, some of which could have contributed to the slightly higher severe complications among children aged 2–59 months. Firstly, data were collected from clinical documentation and caregiver report, without observation or re-examination, to avoid a Hawthorne effect or influencing onward outcomes, enabling ‘real-world’ impact evaluation. This may have led to under- or overestimation of intervention uptake and hypoxaemia prevalence, or missed referral recommendations, particularly if caregivers declined referral, causing potential misclassification bias of hospitalisations without referral. If more referrals were ‘missed’ in intervention arms, proportionate to observed higher referral rates, this could partly explain the higher severe complications observed. Secondly, reliance on routine data sources and caregiver report limited our ability to fully control for residual or unmeasured confounding. Particularly, undetected imbalances of severe illness between arms could have influenced results, either by random allocation of facilities with more severely ill children to intervention arms, or if the intervention modified care-seeking for severe illness towards intervention facilities. Without observation or re-examination, only caregiver reported symptoms were used to assess imbalance; diagnoses could not be used given the intervention's intended effect on classification. Thirdly, low severe complication rates suggest relatively low proportions of severely ill children among the study population, possibly due to bypassing primary care, high care-seeking for milder illness or, though eligible, non-recruitment of severely ill children. Though stakeholders are interested in impact of primary care pulse oximetry on mortality and hospitalisation, future studies may need to consider alternative, less rare, clinical outcomes given the implications on required sample size. Fourthly, given the cluster-level implementation of the intervention at the health facility level, blinding of providers and research assistants was not possible, introducing potential for performance and detection biases inherent to pragmatic trial designs. Lastly, we did not assess using SpO_2_ referral thresholds above 90%, which remains an important question,^15^^,^^37^ but higher severe complications with mild to moderate hypoxaemia (SpO_2_ 90–93%) than normoxaemia reinforce findings from other studies,^9^^,^^37^ highlighting the need for identification and closer management of these children.

In summary, this large-scale pragmatic cluster RCT in India and Tanzania substantially contributes to the sparse evidence on the impact of primary care pulse oximetry, with or without CDSAs, on clinical outcomes of sick children in LMICs. The finding that severe complications were not improved, and were slightly higher among children 2–59 months in intervention arms, should be interpreted cautiously. Higher rates of urgent referrals suggest that pulse oximetry and CDSAs can help healthcare providers identify children with severe disease. However, non-referral of over half of children with hypoxaemia, low rates of referral completion and lack of improvement in hospitalisation and mortality outcomes re-emphasise the major challenges in accessing quality hospital care and designing pragmatic studies to efficiently evaluate impact of context-dependent primary care interventions along the care cascade. Though hypoxaemia prevalence was low among all sick children, substantial evidence associates it with higher morbidity and mortality, and it cannot reliably be detected by clinical signs alone. Collectively these findings suggest that pulse oximetry, CDSAs, and other interventions to support severe illness detection in primary care, must be embedded in wider efforts to implement active follow-up of children with severe illness, strengthen quality as a continuum from primary to hospital care, and address socio-economic determinants of access to care.

## Contributors

FB, HL, HM, SA, TG, FS, KW, VDA, ER, LFB, MR, MEF, HS were responsible for conceptualisation of the study. FB, HL, TG, SC, GL, AM, CF, RM, SA, GA were responsible for the methodology of the study. HM, GM, AM, RM, CF, SL, LM, SM, JM, NM, IEM were responsible for the implementation of the study in Tanzania, and SA, DK, AJ, VB, M, GA, GK in India. MR, MEF, HS, DM, KS were responsible for the intervention implementation, with support from other members of the TIMCI collaborator group. Data was collected by KGMU and IHI research assistants, overseen by GM, SL, CF, DK, AJ. Data management was led and coordinated by HL globally, and by AM, CF, SL, in Tanzania, and DK, AJ, in India. ER and VO were responsible for monitoring of the trial. SC developed the statistical analysis plan with inputs from TG, FB, HL, GL, GM, AM, SL, SA, GA, DK, AJ, VDA, KW and SC performed the statistical analysis with inputs from TG, HL, GL and FB. Interpretation of trial findings was conducted collectively through an in-person workshop involving FB, GM, DK, HL, GL, TG, SC, SL, RM, FS, KS, DM, MR, MEF, MS, EP, SH, VDA, KW and discussion with other members of the consortium and external stakeholders. The original draft of the manuscript was prepared by FB, HL, GL, SC, TG, FS, with review and feedback by all other authors. Visualisations of data presented were prepared by HL, SC and GL with input from TG, FS and FB. Overall oversight was by Principal Investigators KW and VDA globally, HM in Tanzania and SA in India, statistical oversight by TG, and clinically by FB globally, RM in Tanzania and SA in India. FS was responsible for research activity planning and execution globally, with GM responsible in Tanzania and DK in India. KW, VDA, MR, HM, SA and other members of the wider TIMCI collaborator group were responsible for acquiring funds.

## Data sharing statement

De-identified individual children data that underlie the results reported in this manuscript are available through the TIMCI community on Zenodo:

Tanzania: https://zenodo.org/records/12698950.

India: https://zenodo.org/records/14024887.

These datasets are under restricted access until December 2025. During this period, data will be made available upon reasonable request. Researchers interested in accessing the data should submit a detailed request through the TIMCI community on Zenodo, including a methodologically sound proposal. The study team will review all requests to ensure they meet criteria for data sharing and are scientifically valid. From January 2026, the data will become openly accessible under a Creative Commons Attribution 4·0 International license (CC-BY 4·0), with no end date.

The study protocol, statistical analysis plan, and analytic code will be available following publication, with no end date, at https://zenodo.org/communities/timci. These materials will be accessible without restriction.

## Declaration of interests

The authors declare no competing interests. All institutes received funding as part of grant n°2019-35-TIMCI, contributing to salaries of co-authors.

## Appendix A Supplementary data

timci collaborator group

RCT CONSORT 2010 Checklist

TIMCI RCT Cross-CountryStatisticalAnalysisPlan

2024 TIMCI study design

01_RCT_S1

01_RCT_S2

01_RCT_S3

01_RCT_S4

01_RCT_S5

01_RCT_S6
