# Supplementary material for: Effectiveness of introducing pulse oximetry and clinical decision support algorithms for the management of sick children in primary care in India and Tanzania on hospitalisation and mortality: the TIMCI pragmatic cluster randomised controlled trial
Source: eClinicalMedicine. 2025 Jul 3;85:103306. doi: 10.1016/j.eclinm.2025.103306 (PMC12271772; doi:10.1016/j.eclinm.2025.103306)
Supplement: timci collaborator group [file mmc1.docx]

TIMCI Collaborator Group

- Agarwal Girdhar – King George's Medical University (KGMU), India
- Arimi Peter – University of Nairobi (UoN), Kenya
- Awasthi Shally – King George's Medical University (KGMU), India
- Ba Maymouna – PATH, Senegal
- Bandi Vineela – King George's Medical University (KGMU), India
- Beynon Fenella, Swiss Tropical and Public Health Institute (Swiss TPH) / University of Basel, Switzerland
- Bohle Leah F., Swiss Tropical and Public Health Institute (Swiss TPH) / University of Basel, Switzerland
- Bulo Method – PATH, Tanzania
- Cicconi Silvia, Swiss Tropical and Public Health Institute (Swiss TPH) / University of Basel, Switzerland
- Cissé Magib – Mbour hospital, Senegal
- Clemence Zach, PATH, United States
- Cleveley Lisa, Swiss Tropical and Public Health Institute (Swiss TPH) / University of Basel, Switzerland
- Cummings Ray, PATH, United States
- D'Acremont Valérie, Unisanté, University of Lausanne, Switzerland
- Emmanuel-Fabula Mira – PATH, Switzerland
- Faivre Vincent, Unisanté, University of Lausanne, Switzerland
- Faye Mouhamadou Mansour – PATH, Senegal
- Faye Papa Moctar – Cheikh Anta Diop University of Dakar (UCAD), Senegal
- Festo Charles, Ifakara Health Institute (IHI), Tanzania
- Glass Tracy R. , Swiss Tropical and Public Health Institute (Swiss TPH) / University of Basel, Switzerland
- Gupta Kanishka – PATH, India
- Harner-Jay Claudia, PATH, United States
- Horlacher (Shawcross) Megan, PATH, United States
- Horton Susan – University of Waterloo (UoW), Canada
- Ifakara Health Institute (IHI), Tanzania
- Jacob Anmol – King George's Medical University (KGMU), India
- Keitel Kristina, Swiss Tropical and Public Health Institute (Swiss TPH) / University of Basel, Switzerland
- Keith Bonnie, PATH, United States
- Kosgei Rose – University of Nairobi (UoN), Kenya
- Kumar Divas – King George's Medical University (KGMU), India
- Kumar Gaurav, Swiss Tropical and Public Health Institute (Swiss TPH) / University of Basel, Switzerland
- Lalwani Tanya, PATH, United States
- Langet Hélène, Swiss Tropical and Public Health Institute (Swiss TPH) / University of Basel, Switzerland
- Levine Gillian, Swiss Tropical and Public Health Institute (Swiss TPH) / University of Basel, Switzerland
- Lwambura Samwel, Ifakara Health Institute (IHI), Tanzania
- Machoki James – University of Nairobi (UoN), Kenya
- Maiba John, Ifakara Health Institute (IHI), Tanzania
- Makawia Suzan, Ifakara Health Institute (IHI), Tanzania
- Mansi Tyagi – King George's Medical University (KGMU), India
- Martin Gregory, Unisanté, University of Lausanne, Switzerland
- Masanja Honorati, Ifakara Health Institute (IHI), Tanzania
- Masanja Irene, Ifakara Health Institute (IHI), Tanzania
- Masanja Naomi, Ifakara Health Institute (IHI), Tanzania
- Matata Lena, Swiss Tropical and Public Health Institute (Swiss TPH) / University of Basel, Switzerland
- Mhalu Grace, Ifakara Health Institute (IHI), Tanzania
- Miheso Andolo – PATH, Kenya
- Mjungu Deusdedit, PATH, Tanzania
- Mkopi Abdallah, Ifakara Health Institute (IHI), Tanzania
- Moshiro Robert, Muhimbili University Health and Allied Sciences (MUHAS), Tanzania
- Mtebene Ibrahim, Ifakara Health Institute (IHI), Tanzania
- Mugo Mercy – University of Nairobi (UoN), Kenya
- Ndiaye Ousmane – Cheikh Anta Diop University of Dakar (UCAD), Senegal
- Ngari Kevin – University of Nairobi (UoN), Kenya
- Ngutu Mariah – University of Nairobi (UoN), Kenya
- Njiri Francis – University of Nairobi (UoN), Kenya
- Norris Martin, Swiss Tropical and Public Health Institute (Swiss TPH) / University of Basel, Switzerland
- Odek Olgah – PATH, Tanzania
- Oliveira Vânia, Swiss Tropical and Public Health Institute (Swiss TPH) / University of Basel, Switzerland
- Onah Michael – University of Waterloo (UoW), Canada
- Orschulko Anja, Swiss Tropical and Public Health Institute (Swiss TPH) / University of Basel, Switzerland
- Oviedo Dickens – PATH, Kenya
- Pantjushenko Elena, PATH, United States
- Quintanar Solares Manjari, PATH, United States
- Rajaratnam Julie, PATH, United States
- Rastogi Tuhina – King George's Medical University (KGMU), India
- Reus Elisabeth, Swiss Tropical and Public Health Institute (Swiss TPH) / University of Basel, Switzerland
- Ruffo Mike – PATH, Switzerland
- Schär Fabian, Swiss Tropical and Public Health Institute (Swiss TPH) / University of Basel, Switzerland
- Schaufelberger Sylvain, Unisanté, University of Lausanne, Switzerland
- Sharma Kovid – PATH, India
- Shauri Janet – PATH, Kenya
- Smith Lisa, PATH, United States
- Sougou Ndèye Marème – Cheikh Anta Diop University of Dakar (UCAD), Senegal
- Storey Helen, PATH, United States
- Tan Rainer, Unisanté, University of Lausanne, Switzerland
- Thabard Julian, Unisanté, University of Lausanne, Switzerland
- Thiongane Aliou  – Cheikh Anta Diop University of Dakar (UCAD), Senegal
- Tine Jean Augustin Diégane – Cheikh Anta Diop University of Dakar (UCAD), Senegal
- Vonlanthen Alan, Unisanté, University of Lausanne, Switzerland
- Wyss Kaspar, Swiss Tropical and Public Health Institute (Swiss TPH) / University of Basel, Switzerland
