# Supplementary material for: Effectiveness of introducing pulse oximetry and clinical decision support algorithms for the management of sick children in primary care in India and Tanzania on hospitalisation and mortality: the TIMCI pragmatic cluster randomised controlled trial
Source: eClinicalMedicine. 2025 Jul 3;85:103306. doi: 10.1016/j.eclinm.2025.103306 (PMC12271772; doi:10.1016/j.eclinm.2025.103306)
Supplement: TIMCI RCT Cross-CountryStatisticalAnalysisPlan [file mmc3.pdf]

STATISTICAL ANALYSIS PLAN

TIMCI: Tool for Integrated Management of Childhood Illness

Cross-country Randomised Control Trial

**Date prepared**	05.04.2023
**SAP Version**	1.0
**Protocol Version**	2.4
**Study registration number (registry)**	NCT04910750 (clinicaltrials.gov)

Swiss TPH
Swiss Tropical and Public Health Institute
Schweizerisches Tropen- und Public Health-Institut
Institut Tropical et de Santé Publique Suisse
Associated Institute of the University of Basel

ABBREVIATIONS

**CI**	Confidence interval
**CDSA**	Clinical Decision Support Algorithm
**CG**	Caregiver
**CONSORT**	Consolidated Standards of Reporting Trials
**DMC**	Data Monitoring Committee
**HC**	Health Center
**HCP**	Health Care Provider
**ICC**	Intracluster Correlation Coefficient
**ID**	Identification
**IHI**	Ifakara Health Institute
**IQR**	Interquartile Range
**IMCI**	Integrated Management of Childhood Illness
**LMIC**	Low – and middle – income countries
**KGMU**	King George's Medical University
**RA**	Research Assistant
**RCT**	Randomised Controlled Trial
**SAP**	Statistical Analysis Plan
**SD**	Standard Deviation
**SPA**	Service Provision Assessment
**Swiss TPH**	Swiss Tropical and Public Health Institute
**TIMCI**	Tool for Integrated Management of Childhood Illness
**WHO**	World Health Organization

TABLE OF CONTENTS

TABLE OF CONTENTS **3**
1 Administrative Information **5**
1.1 Document Scope 5
1.2 Document History 5
1.3 Roles and Responsibilities 6
1.4 Signatures 6
2 Introduction **7**
2.1 Background and Rationale 7
2.2 Objectives 7
3 Study methods **8**
3.1 Study Design 8
3.1.1 Overview 8
3.1.2 Study procedures 8
3.1.3 Study intervention 9
3.1.4 Outcomes 9
3.2 Randomisation 10
3.3 Blinding 10
3.4 Sample Size 11
3.5 Framework 11
3.6 Statistical interim Analysis and stopping Guidance 11
3.7 Timing for Analysis 12
3.8 Timing for Baseline and Outcome Assessments 12
4 Statistical Principles **12**
4.1 Confidence Intervals and p-Values 12
4.2 Intervention adherence and Protocol Deviations 13
4.3 Analysis Populations 13
5 Study Population **14**
5.1 Screening 14
5.2 Eligibility 14
5.3 Recruitment 14
5.4 Withdrawal/Follow-up 15
5.5 Baseline Participant Characteristics 15
6 Analysis **15**
6.1 Outcomes Definitions 15
6.1.1 General definitions 16
6.1.2 Health, hospitalisation and referral outcomes 17
6.1.3 Antibiotic-related outcomes 19
6.1.4 Antimalarial-related outcomes 20
6.1.5 Hypoxaemia-related outcomes – only in intervention arms 21

6.1.6 Follow-up outcomes 21
6.2 Analysis Methods 23
6.2.1 Analytical Methods 23
6.2.2 Adjustment for Covariates 23
6.2.3 Test of Assumptions, Actions to be taken 23
6.2.4 Pre-planned Sensitivity Analyses 23
6.2.5 Pre-specified Subgroups Analysis 24
6.3 Missing Data 25
6.4 Additional analyses 25
6.5 Harms 25
6.6 Statistical Software 25
7 References 25
7.1 SOPs, study-specific Documents 25
7.2 External references 26
8 APPENDIX A 26
Figure 1) Recommended standardised age disaggregation groups for data analysis by life stage [6]. 27

1 ADMINISTRATIVE INFORMATION

1.1 Document Scope

This document provides a detailed description of the methodologies that will be followed, as closely as possible, when analysing and reporting results from TIMCI RCT study. The planned analysis detailed in this document is in compliance with that briefly specified in the TIMCI RCT protocol, except if otherwise detailed.

The purpose of this Statistical Analysis Plan is to:

- Ensure that the analysis is appropriate for the aims of the study, reflects good statistical practice in general, and minimises bias.
- Ensure that the analyses performed are consistent with the conditions of the protocol.
- Explain in detail how the data will be handled, covariates derived and analysed to ensure transparency and reproducibility, including enabling others to perform the actual analysis in the event of sickness or other absence.
- Protect the project by helping it keep to timelines and within scope.

Additional exploratory or auxiliary analyses of data not specified in the protocol or this document are permitted but fall outside the scope of this Statistical Analysis Plan. Where analyses are presented which are not included in the SAP, these will be clearly indicated as such along with a justification as to their inclusion.

The analysis strategy will be made available if required by journals or referees when the main papers are submitted for publication. Additional analyses suggested by reviewers or editors will, if considered appropriate, be performed in accordance with the analysis plan, but if reported, the source of such post-hoc analyses shall be declared.

This document is written following the guidelines outlined in Gamble et al. [1]

1.2 Document History

Statistical Analysis Plan Version	Protocol Version	Section number(s) changed	Description of changes	Date	By whom
1.0	2.4	-	New document	05/04/2023	Silvia Cicconi

1.3 Roles and Responsibilities

Name	Affiliation	Role in the study
**Ms. Silvia Cicconi**	Swiss TPH	Trial Statistician
**Dr. Tracy Glass**	Swiss TPH	Senior Statistician
**Dr. Fenella Beynon**	Swiss TPH	Clinical Research Scientist
**Dr. Hélène Langet**	Swiss TPH	Data Manager
**Prof. Kaspar Wyss**	Swiss TPH	PI
**Prof. Valerie D'Acremont**	Swiss TPH	PI
**Dr. Gaurav Kumar**	Swiss TPH	Country Coordinator India
**Dr. Lena Matata**	Swiss TPH	Country Coordinator Tanzania
**Prof. Shally Awasthi**	KGMU	India PI
**Dr. Divas Kumar**	KGMU	India Study Coordinator
**Dr. Girdhar Agarwal**	KGMU	India Senior Statistician
**Anmol Jacob**	KGMU	India Data Manager
**Dr. Honorati Masanja**	IHI	Tanzania PI
**Dr. Grace Mhalu**	IHI	Tanzania Study Coordinator
**Dr. Robert Moshiro**	IHI	Tanzania Clinical Scientist
**Dr. Abdallah Mkopi**	IHI	Tanzania Senior Research Scientist
**Mr. Samwel Lwambura**	IHI	Tanzania Data Manager

1.4 Signatures

Name	Responsibility	Signature	Date
Silvia Cicconi	Author	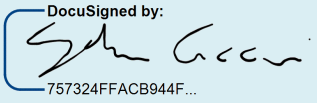	26-May-2023 | 13:30:41 CEST
Tracy Glass	Statistical reviewer	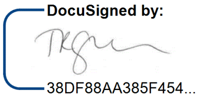	30-May-2023 | 09:56:06 CEST
Fenella Beynon	Clinical reviewer	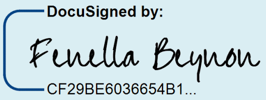	30-May-2023 | 08:41:53 CEST

Kaspar Wyss	Reviewer	DocuSigned by: Kaspar Wyss 2AAD51657B2149B...	26-May-2023 | 03:34:09 PDT
Valerie D'Acremont	Reviewer	DocuSigned by: Valerie D'Acremont EBC5406609F2400...	26-mai-2023 | 14:04:03 PDT
Shally Awasthi	Reviewer	DocuSigned by: Shally Awasthi 5BA2FF1C2E1941A...	01-Jun-2023 | 03:55:17 PDT
Honorati Masanja	Reviewer	DocuSigned by: [illegible] 728BE860CB8A417...	31-May-2023 | 05:03:21 PDT

2 INTRODUCTION

2.1 Background and Rationale

Despite progress in reducing child mortality in the last few decades, 5.2 million children under five years of age died of preventable causes. Strengthening systems to identify and appropriately treat sick children, alongside health prevention and promotion activities, is critical to reduce under-five mortality. The Integrated Management of Childhood Illness (IMCI) guidelines, launched in 1995 and now adopted by over 100 countries, responded to the need to systematise the implementation of evidence-based health interventions for children under five in primary care and the community [2]. Yet despite the intention of IMCI to have high sensitivity for detection of severe disease, non-adherence by health workers and intrinsic problems of guidelines based on clinical signs alone are causes of poor identification and management of sick children. The Tools for the Integrated Management of Childhood Illness (TIMCI) project seeks to address these issues by introducing pulse oximetry and clinical decision support algorithms (CDSAs) to strengthen guideline implementation and accuracy in order to improve quality of care and reduce morbidity & mortality of children under five years of age.

Please refer to the section Background and rationale of the Protocol for more information.

2.2 Objectives

The overall goal of the TIMCI project is to reduce morbidity and mortality in sick children attending primary care facilities, while supporting the rational and efficient use of diagnostics and medicines by health care providers (HCP). The implementation research component of the project seeks to generate evidence on the health impact, operational priorities, cost and cost-effectiveness of introducing pulse oximetry, alone or embedded into a CDSA, into primary

care in LMICs, for children 0 – 59 months of age, to facilitate national and international decision-making on scale-up.

3 STUDY METHODS

3.1 Study Design

3.1.1 Overview

The TIMCI study is an international project involving diverse health systems and communities in India, Kenya, Senegal and Tanzania. The study setting includes facilities providing primary care services, including outpatient settings within larger health centres in addition to the more traditionally-labelled primary care facilities.

As part of the evaluation in India and Tanzania, a pragmatic, parallel group, superiority cluster randomised controlled trial will compare health outcomes between pulse oximetry and control (1:1) in India, and pulse oximetry plus CDSA, pulse oximetry alone and routine care (1:1:1) in Tanzania. More details of intervention can be found in Section 3.1.3 and the Intervention section of the Protocol.

Clustering is at the facility level and it has been chosen to: avoid contamination that would occur if randomisation was at the individual level; avoid introducing different processes within one facility; and enable evaluation of effectiveness in real-world settings.

The assessment of the health impact of the intervention will be complemented by embedded mixed methods sub-studies to evaluate other key components and gain a deeper understanding of the implementation mechanisms and context. These studies include a modified service provision assessments (SPAs) and qualitative studies. Data from these studies will be triangulated in order to generate in-depth insights into implementation. Please refer to the Study Design section of the Protocols for more details.

3.1.2 Study procedures

Research assistants (RAs) will screen and recruit participants in the waiting area prior to consultation (Day 0). Following children's caregiver informed consent, the RA will record participant name, contact details, sociodemographic details and reason for attendance prior to consultation. In case of critical and emergency cases, children will be seen immediately by the HCP. The clinical consultation will be conducted according to the trial arm of the facility allocation.

After consultation, the RA will extract information from clinical records, the caregiver and/or medical records and will document them in the database. This information includes final diagnosis, oxygen saturation and results of laboratory investigations, prescribed medication, referral or follow-up advice and usage of pulse oximeter and/or CDSA.

At Day 7 and Day 28 the RA will conduct follow-up, remotely when possible.

For caregivers who can provide a phone number at the day of enrolment, Day 7 and Day 28 follow-ups will be conducted by phone. If the initial phone call fails and the phone number is valid, at least three attempts will be made on subsequent three days at different times of the day to try to reach the caregiver. If the phone number is not valid, no other call attempts will be made and alternative follow-up options will be initiated if consented for. Except during the initial phone call, follow-up can be carried out with a caregiver other than the one who gave

consent at the day of enrolment if this person is able to provide information about the child. For caregivers who cannot provide a phone number at the day of enrolment, community mechanisms will be relied on to arrange a call with the caregiver through a community focal person, e.g. a community health worker (CHW) or a community leader. To this aim, CHWs and community leaders in the targeted study areas will be asked for their consent to have their phone numbers shared with RAs in order to contact caregivers without a phone for their Day 7 and Day 28 follow-ups. Independently on whether they provided a phone number or not, caregivers will be given the alternative option to consent for in-person Day 7 follow-up that will have to be conducted as soon as possible after all remote attempts failed. In-person Day 7 follow-ups could be conducted either at the caregiver's household or another specified community location.

If Day 7 follow-up has been unsuccessful, Day 28 follow-up will still be attempted. During both Day 7 and Day 28 follow-ups, a structured questionnaire will be administered to assess the primary and secondary outcomes, including details of any government or private hospital admission. In case of non-recovery, if the child is still at home, the study team will advise the family to return to the PHC clinic for a follow-up consultation.

3.1.3 Study intervention

- Pulse oximetry embedded into CDSA
  - Pulse oximeters, with guidance (integrated into CDSA), training and mentorship
  - Tablet-based CDSAs, with guidance, training and mentorship
  - Refresher training on latest national / IMCI guidelines integrated with pulse oximetry and CDSA training
- Pulse oximetry with paper job aid
  - Pulse oximeters, with guidance (pulse oximetry specific job-aid, plus adapted IMCI chart book), training and mentorship
  - Refresher training on latest national / IMCI guidelines integrated with pulse oximetry training
- Control (routine care)
  - Refresher training on latest national / IMCI guidelines

3.1.4 Outcomes

This section provides an overview of the outcomes as listed in the protocol.

Primary outcomes

- Proportion of children with a severe complication by Day 7
- Proportion of children admitted to hospital within 24 hours of the Day 0 consultation and as a result of a referral (this is a proxy for 'appropriate referral' of children)

Secondary outcomes

- Proportion of children with a severe complication by Day 28
- Proportion of children cured at Day 7 follow-up
- Proportion of children referred by a primary healthcare provider to a higher level of care at Day 0 consultation
- Proportion of children who completed referral as reported at Day 7 follow-up

Other outcomes of interest – hypoxaemia (in intervention arms only)

- Proportion of children with severe (SpO2 <90%), moderate (SpO2 90 – 91%) and mild (SpO2 92 – 93%) hypoxaemia, adjusted for sites at high altitude
- Proportion of children with hypoxaemia (according to differing cut-offs) with severe complication

- Proportion of children with severe hypoxaemia not meeting any other clinical criteria for severe disease
- Proportion of children referred with hypoxaemia who receive oxygen at hospital

Other outcomes of interest – referral and follow-up

- Proportion of children with non-severe disease referred to a higher level of care on Day 0
- Average length of stay (in days) of children admitted to hospital
- Proportion of children attending scheduled follow-up at the same facility by Day 7
- Proportion of children presenting for unscheduled follow-up to any health facility by Day 7

Other outcomes of interest – antimicrobial prescription

- Proportion of children prescribed an antibiotic at Day 0
- Proportion of children prescribed a diagnosis-appropriate antibiotic
- Proportion of febrile children tested for malaria at Day 0
- Proportion of malaria positive children prescribed an antimalarial
- Proportion of malaria negative children prescribed an antimalarial
- Proportion of untested children prescribed an antimalarial

Other factors for exploratory analyses

- Additional socio-demographic characteristics, urban / rural location, distance from health facility, key household indicators such as maternal age and education
- Main complaint / symptom categories (including cough / difficulty breathing, fever, diarrhoea and other) and time since onset of symptoms
- Alternative hypoxaemia cut-offs
- Health care provider severity classification
- Facility and health system factors – type of facility, health care provider qualification and previous training

These exploratory analyses follow outside the scope of this statistical analysis plan and they will not detailed further here but in a separate document.

3.2 Randomisation

The allocation sequence was generated separately for each country, stratified by facility type (dispensary/health center) and location (urban/rural) in Tanzania; by facility type (primary healthcare centres (HC)/community health centers) and district (Deoria, Sitapur, Unnao) in India. The target number of facilities were randomly selected from all eligible facilities and allocated to the trial arms. The remaining eligible facilities were retained in a back-up list for later allocation (using the same procedure) should the need arise (e.g. in case of facility exclusion at study start, or in case of need to add additional clusters in the event of recalculation of sample size). Given the cluster design, concealment will occur only at the stage of allocation of facilities, which will be conducted centrally and distributed to study sites. Participants will receive the intervention based on the facility they attend.

The allocation sequence generation was carried out by a statistician, independent from the study, prior to the recruitment of the first patient.

3.3 Blinding

This is an open-label study.

3.4 Sample Size

The sample size for the pragmatic RCT is calculated separately for each country, based on the following assumptions:

- power of 80%
- alpha level of 0.05 per arm
- intra-cluster correlation coefficient (ICC) of 0.001
- 1.1% severe complications in the control arm
- 1.5% appropriate hospitalisations (within 24 hrs of Day 0 and as a results of referral) in the control arm

In India, initial sample size calculation for the three arm trial was based on having the power to detect a 30% or greater decrease in severe complication and 30% or greater increase in appropriate hospitalisation for each arm compared to control with 40 cluster per arm. This assumes average recruitment of 510 children per cluster over the 12 month period (total 40800), with an average of 110 per CHC per month and 20 children per PHC, assuming an average facility ratio CHC : PHC of 1:3. Following the decision, based on the pilot, not to introduce the CDSA, facilities were reallocated to the remaining two arms up to 60 facilities per arm.

In Tanzania, the study would have the power to detect a 30% or greater decrease in severe complication and a 30% or greater increase in appropriate hospitalisation for each arm compared to control with 22 clusters per arm. This assumes average recruitment of 1680 children per cluster over the 12 month period (total 110880), with an average of 350 children per health centre per month and 70 children per dispensary per month, assuming an average facility ratio health center : dispensary of 1:3.

3.5 Framework

TIMCI is a superiority study aiming to show the benefits, in terms of mortality and morbidity in children 0-59 months, of introducing pulse oximetry with updated IMCI chart booklet plus pulse oximetry job aid or pulse oximetry embedded into a CDSA, compared to routine care in primary health care facilities. The primary outcomes will be evaluated according to hierarchy of importance as described in Section 4.1.

3.6 Statistical interim Analysis and stopping Guidance

An interim analysis is planned to be conducted three months after the start of the study to assess recruitment rate, follow-up and sample size assumptions. The Data Monitoring Committee (DMC) will review the results and make recommendations to the Research Steering Committee (RSC) as to whether:

- the sample size calculation should be adjusted based on estimated values in the control groups, as well as facility inclusion and recruitment;
- the primary outcome measures should be revised due to a large percentage of missing values;
- the study should be terminated prematurely due to inability to achieve its objectives.

A Data Monitoring Committee will be formed to monitor the progress of the trial. Further details are provided in the DMC charter [B]. Of note, no formal stopping rules are applied, and the criteria for the strength of evidence is left to the judgement of the DMC.

3.7 Timing for Analysis

An interim analysis will be triggered after three months from the study start. Data will be analysed after all children recruited in the first three months reach Day 7 follow-up and after data cleaning processes. The data export will include all data collected on these children up to the date of the export. Any interim analyses will be performed separately for Tanzania and India.

The final analysis will take place once all children have received a minimum of 28 days of follow-up. A pooled cross-country analysis will be conducted as well as individual country analyses.

3.8 Timing for Baseline and Outcome Assessments

Baseline is defined as the date of enrolment (Day 0) and time will be measured from this point.

Follow-up will occur 7 days and 28 days after Day 0. As defined in the protocol, no time window applies to Day 7 follow-up and a window of + 7 days applies to Day 28 follow-up. Refer to "Participants timeline" section of the protocol for more details.

The components (i.e. death and hospitalisation) required for the analysis of the primary outcomes will be assessed at both Day 7 and Day 28 follow-ups. For the main analysis, date of death and date of hospitalisation will be used irrespective of when these information are collected.

4 STATISTICAL PRINCIPLES

4.1 Confidence Intervals and p-Values

For the final analysis of the primary endpoints, a hierarchical fallback procedure will be used, which is a closed test procedure using a weighted Bonferroni [3, 4]. This method recycles unspent significant levels to test subsequent hypotheses with a pre-specified hypothesis sequence [5]. It uses alpha propagation making it more powerful that the Bonferroni method.

The primary outcomes will be evaluated according to hierarchy of importance as follows:

H_1: There is no difference between the experimental arm and control arm with respect to severe complications by day 7.

H_2: There is no difference between the experimental arm and control arm with respect to appropriate hospitalisation.

First, the null hypothesis H_{12}, which is that neither H1 nor H2 are significant, will be tested. The null hypothesis H_{12} will be rejected if either $p1 \leq \alpha1$ or $p2 \leq \alpha2$, where α is equally distributed between the endpoints i.e. $\alpha1=0.025$ and $\alpha2=0.025$.

Failure to reject the null hypothesis H_{12} we shall conclude the study to be negative as neither of the co-primary outcomes were significant and no further analysis will be performed. If the null hypothesis H_{12} is rejected, then H_1 and H_2 will be tested. If $p2 \leq \alpha2$ then we reject H_2 and test H_1 as $p1 \leq \alpha$. If $p2 > \alpha2$ then we do not reject H2 and test H1 as $p1 \leq \alpha1$.

This procedure will be followed for each of the two experimental arms compared to the control arm. All outcomes will be evaluated regardless of the outcome of the first evaluation. For each intervention arm, the overall trial will be interpreted as positive if either primary outcome is positive compared to control.

Results estimates of primary and secondary outcomes will be presented along with the associated two-sided 95% confidence intervals, for both final analysis and analyses performed for DMC meetings. No adjustments will be made for the interim analysis and any other analysis carried out for the DMC review since they will not be conducted with the purpose of efficacy evaluation.

4.2 Intervention adherence and Protocol Deviations

While in India pulse oximeter was indicated for all children, in Tanzania, pulse oximeter was indicated for all young infants and for older children with cough/difficult breathing or red/yellow IMCI classification. Cough/difficulty breathing and red/yellow IMCI classification are defined two ways, based on signs and symptoms as reported by the caregiver pre-consultation or on diagnosis made by the healthcare provider during the consultation. These definitions are detailed in a separate document and both will be used, separately, to assess intervention adherence.

Adherence will be evaluated separately for the two intervention arms on the basis of pulse oximeter and CDSA use when indicated.

- Pulse oximetry adherence will be evaluated primarily using information on SpO2 values from clinical records. Pulse oximetry will be considered used if the RA has found any SpO2 recording (value readable or unreadable). Pulse oximetry will be considered not used if the RA did not find any recorded SpO2. Adherence will be additionally evaluated based on pulse oximetry use as reported by the caregivers.

- CDSA adherence will be evaluated by comparing the number of children enrolled and the number of children recorded into the CDSA database. These data will be used to explore further and in more details adherence to the CDSA, but outside the scope of this SAP and it will be detailed in a separate document.

Adherence will be reported overall and weekly by facility.

The following information will be summarised by arm to evaluate deviations to the study protocol:

- Number and percentage of caregivers for which no Day 7 follow-up was attempted.
- Number and percentage of caregivers for which no Day 28 follow-up was attempted.

More general deviations will be recorded by the study team in a study deviation log and categorised into minor and major.

4.3 Analysis Populations

All analyses will follow the intention to treat (ITT) principle, retaining facilities and participants in their initially randomised groups irrespective of the intervention received or any protocol violations. However, clusters provided from the start of the trial with a different intervention package than the one they were randomised to will be analysed according to the arm of the intervention they delivered.

A complete case set will also be defined, including only participants whose follow-up information are available (i.e. at least one follow-up was conducted). Analysis of the primary outcomes will be performed on both the ITT and the complete case set for final analysis and analyses for the DMC review.

No safety set will be defined but safety information will be reported overall and by use of intervention as defined in section 4.2.

5 STUDY POPULATION

5.1 Screening

No specific screening data will be captured, aside from those required to assess children's eligibility for the study (see section 5.2). The number of potential participants screened for the study will be presented in a CONSORT diagram by intervention arm.

5.2 Eligibility

The following are inclusion and exclusion criteria that determine facility and child eligibility:

Facility inclusion:

- Consenting government-designated healthcare facilities within the selected geographical areas of each country
- Providing curative primary care services for children 0 – 59 months of age
- Oxygen available or referral mechanism in place with oxygen available at a higher level facility
- Electricity available (from any source with continuous or intermittent supply)

Facility exclusion:

- Attending to fewer than 20 sick children per month (based on the prior 12 month average)
- Already using pulse oximetry as a routine part of outpatient-based consultations of children 0 – 59 months
- Selected to be part of a major child health programmatic or research intervention during the study period likely to significantly affect the primary outcome
- Inaccessible to the study team (e.g. due to weather conditions or security issues) for significant parts of the year.

Individual child inclusion:

- Children 0 – 59 months for whom caregivers provide consent
- Consulting for an illness, or reported to be unwell when attending for a routine visit (e.g. vaccination, growth or chronic disease monitoring)

Individual child exclusion:

- Children in the immediate post-natal period or first day of life
- Attending for a consultation related to trauma only (including new and follow-up presentations for burns, injuries, wounds)
- Admitted within an inpatient part of the facility (including neonates delivered at the facility admitted with their mother)
- Enrolled in the study within the preceding 28 days at any study facility

5.3 Recruitment

The number of participants enrolled will be presented in a CONSORT flowchart by intervention arm, together with the number of potential participants not recruited due to the child's eligibility

failure, caregiver's refusal to consent or caregiver being underage. Reasons for eligibility failure will be summarised.

Further enrolment figures will be presented by facility type, location and district and a graph will be produced showing monthly and cumulative recruitment.

Additionally, the percentage of eligible children (no. eligible/no. screened) and the percentage of recruited children (no. recruited/no. eligible) will be provided by intervention arm.

5.4 Withdrawal/Follow-up

The number of children for which follow-up was conducted will be presented in the CONSORT diagram by intervention arm for each of the scheduled assessment time point (Day 7 and Day 28). The number of children withdrawn in between follow-ups will also be summarised and reasons provided if available.

5.5 Baseline Participant Characteristics

Socio-demographic data of children enrolled and their caregivers will be summarised. Additional characteristics of interest, such as danger signs and symptoms as reported by the caregiver, will be presented as part of baseline summaries. Baseline data will be presented by intervention arm using median and associated interquartile range (IQR) if continuous and as counts and percentages if categorical. Summary statistics will be of non-missing values, with the number (%) of missing values given if data are not complete. Assessment will be made for baseline imbalances between the randomisation groups by visual inspection only and no formal testing will be performed.

For the baseline summaries, age of children will be reported as a continuous variable in months but also disaggregated in age groups. In the database, the age is estimated based on recommended standardised age disaggregation groups [6] with a different level of accuracy depending on the information provided by the caregiver. Based on age estimates, children are further categorised into young infants (<60 days) and children 60days-59 months. More details are provided in Appendix A.

6 ANALYSIS

Analyses will follow Consolidated Standards of Reporting Trials (CONSORT) guidelines [10] and will be performed and reported overall and by country.

Outcomes will be presented separately for young infants (<60 days) and older children (60 days-59 months), the malaria-related outcomes will be presented only on older children.

Results of interim analyses and DMC analyses will be presented overall in the open report and by intervention arm in the closed report.

6.1 Outcomes Definitions

This section presents general definitions, applicable to multiple outcomes, and a detailed definition of each primary and secondary outcome.

Outcomes definitions are grouped by topic rather than primary and secondary outcomes.

6.1.1 General definitions

Day 7 / Day 28 assessment

Day 7 / Day 28 assessment refers to information reported by the caregiver after Day 0 and recorded in Day 7 / Day 28 forms. Data collected as part of unscheduled visits are also integrated in these forms.
If multiple successful follow-up assessments for the same child are recorded within Day7 or Day28 databases, the most recent follow-up assessment within each database will be used. To limit potential recall bias, assessments conducted far beyond the expected follow-up time will be dropped. This will be 30 and 60 days from Day 0 for Day 7 and Day 28 respectively.

Death

A child will be considered deceased if, from Day 7 / Day 28 assessment, his/her status at the time of follow-up is "deceased". In case of implausible death date (i.e. prior to enrolment date), death will be handled as follow:

- if death date is recorded at Day7 follow-up, death will be assumed to be within 7 days of Day0
- if death date is not recorded at Day7 follow-up but only at Day28 follow-up, death will be assumed to be after 7 days of Day0 but within 28 days.

Hospitalisation

A child will be considered hospitalised if, from Day 7 / Day 28 assessment, he/she went to a hospital, was admitted overnight or if his/her status at the time of follow-up is "hospitalised". In case of implausible hospitalisation date (i.e. prior to enrolment date), hospitalisation will be handled as follow:

- if hospitalisation date is recorded at Day7 follow-up, hospitalisation will be assumed to be within 7 days but after 24hrs of Day0
- if hospitalisation date is not recorded at Day7 follow-up but only at Day28 follow-up, hospitalisation will be assumed to be after 7 days of Day0 but within 28 days.

Hospitalisation within 24hrs of Day0

For the purpose of primary analysis, a child will be considered admitted to hospital 24hrs of Day0 consultation if the date of hospitalisation is the same as Day0 date or is one day after Day0 date.

Referral

A child will be considered to be referred (either to a hospital or to an inpatient part of a larger primary healthcare facility) if, at Day0, the caregiver confirms that the child was urgently referred or the research assistant confirms that an urgent referral advice was recorded for the child. If urgency is unknown from both sources, referral will be assumed to be urgent.[1]

Antibiotics

Antibiotics will be defined in accordance with the WHO antibiotic point prevalence survey (PPS) methodology [7, 8, 9] and therefore only antibacterials for systemic use will be taken into account.

---

[1] Urgency, as reported by the caregiver, was only collected in Tanzania. For India, urgent referrals will be defined based on the registry alone.

SpO2 values

SpO2 should be recorded during the Day0. For the outcomes, the following measures will be taken for spurious and missing SpO2 values:

- If SpO2 value is not available, the child will be assumed normoxaemic
- If 70 ≤ SpO2 < 90%, the child will be assumed (with accuracy) to have severe hypoxaemia
- If 40 ≤ SpO2 < 70,% the child will be assumed (with low accuracy) to have severe hypoxaemia
- If SpO2 < 40%, the value will be considered spurious (e.g. due to incorrect data entry) and treated as not available, i.e. the child will be assumed normoxaemic.

Additionally, a description of SpO2 values distribution will be provided based on the following cut-offs:

- 95% ≤ SpO2 < 100%
- 94% ≤ SpO2 < 95%
- 92% ≤ SpO2 < 94%
- 90% ≤ SpO2 < 92%
- 70% ≤ SpO2 < 90% (severe hypoxaemia, accuracy)
- 40% ≤ SpO2 < 70% (severe hypoxaemia, but low accuracy)
- Spurious (0% ≤ SpO2 < 40%)
- Missing

In India, where three SpO2 measures were collected, only the first one will be used.

Mild hypoxaemia

92% ≤ SpO2 < 94%

Moderate hypoxaemia

90% ≤ SpO2 < 92%

Severe hypoxaemia

SpO2 < 90%

6.1.2 Health, hospitalisation and referral outcomes

6.1.2.1 Proportion of children with a severe complication by Day 7

Severe complication is defined as death or secondary hospitalisation. Secondary hospitalisation refers to any delayed hospitalisation (occurring at any point greater than 24hrs after the Day 0 consultation) and any hospitalisation occurring without a referral.

Information on death and hospitalisation will be taken, in order, from:

- Day7 follow-up assessment
- If Day7 follow-up assessment is missing, Day28 follow-up assessment

Death will be counted in the numerator if death date is within 7 days of Day0 (7th day included). Hospitalisation will be counted in the numerator if the child was not referred at Day0 or hospital admission date is within 7 days of Day0 (7th day included) but after 24hrs of Day0.

The denominator will be all children recruited. Children for whom no death and hospitalisation data are available or withdrew prematurely from the study will be assumed to be alive and not hospitalised by Day7.

6.1.2.2 Proportion of children with a severe complication by Day 28

Severe complication is defined as per the outcome above.

Information on death and hospitalisation will be taken, in order, from:

- Day7 follow-up assessment
- If Day7 follow-up assessment is missing or no outcomes were reported, Day28 follow-up assessment
- If both Day7 and Day28 assessments are missing or no outcomes were reported, unscheduled visits (deaths only)

Death will be counted in the numerator if death date is within 28 days of Day0 (28th day included). Hospitalisation will be counted in the numerator if the child was not referred at Day0 or hospital admission date is within 28 days of Day0 (28th day included) but after 24hrs of Day0.

The denominator will be all children recruited. Children for whom no death and hospitalisation data are available or withdrew prematurely from the study will be assumed to be alive and not hospitalised by Day28.

6.1.2.3 Proportion of children admitted to hospital within 24 hours of Day 0 consultation and as a result of a referral

Information on hospitalisation will be taken, in order, from:

- Day7 follow-up assessment
- If Day7 follow-up assessment is missing, Day28 follow-up assessment

Hospitalisation will be counted in the numerator only if the child received a referral advice (as per definition above) at Day0 and hospital admission date is within the end of the following day. The denominator will be all children recruited.

Additionally, the proportion will be calculated and reported using only children who were referred as denominator.

6.1.2.4 Average length of stay (in days) of children admitted to hospital

Length of stay is defined as hospitalisation duration in days as reported by the caregiver at Day 7 and Day 28. If a child is hospitalised twice, the first hospitalisation will be used and second hospitalisations will be reported separately. The denominator will be all hospitalised children.

6.1.2.5 Proportion of children referred by a primary healthcare provider to a higher level of care at Day 0 consultation

All children who received a referral advice (as per definition above) at Day0 will be counted in the numerator. The denominator will be all children recruited.

6.1.2.6 Proportion of children who completed referral as reported at Day 7 follow-up

A referral will be considered completed if a child attended a hospital, whether he/she was admitted or not. Children will be counted in the numerator if they received a referral advice (as per definition above) and their caregiver reported to have sought care in a hospital and

the follow-up date is within 7 days of Day 0, considering a + 3 days window (10th day included). The denominator will be all children recruited.

Additionally, the proportion will be calculated and reported using only children who were referred as denominator.

Children for whom no Day7 data are available or withdrew prematurely from the study will be assumed not attending a hospital by Day7.

6.1.2.7 Proportion of children with non-severe disease referred to a higher level of care on Day 0

Children with non-severe disease who received a referral advice (as per definition above) at Day0 will be counted in the numerator. The denominator will be all children recruited. Non-severe disease will be defined in a separate document.

6.1.2.8 Proportion of children cured at Day 7 follow-up

A child is defined as cured if he/she recovers from illness. A child will be counted in the numerator as cured if his/her status at the time of Day 7 follow-up is at home “well, completely recovered” and the follow-up date is within 7 days of Day 0, considering a + 3 days window (10th day included). The denominator will be all children recruited.

Children for whom no Day7 data are available or withdrew prematurely from the study will be assumed cured by Day7.

6.1.3 Antibiotic-related outcomes

6.1.3.1 Proportion of children prescribed an antibiotic at Day 0

Information on antibiotics prescription are recorded by the RA on Day 0, after the clinical consultation. The data collection tool is structured in a way to facilitate the reporting of antibiotics, starting with single-drug questions for the most common/important ones to drop-down lists for other antibiotics/treatments and free text. All options will contribute to determine whether an antibiotic prescription was given. A child will be considered prescribed with an antibiotic if:

- “Yes” was selected for at least one single-drug question
- “No” was selected for all single-drug questions but one or more antibiotics were selected from the antibiotics/treatments drop-down list
- “No” was selected for all single-drug questions, antibiotics were not selected from the other antibiotics/treatments drop-down list but antibiotics were listed in the free text field (conditional to feasibility of assessing free text)

All children recruited will be counted in the denominator.

6.1.3.2 Proportion of children prescribed a diagnosis-appropriate antibiotic

Appropriateness of antibiotic prescription in relation to diagnosis will be evaluated as:

- Proportion of children who received a diagnosis for which a systemic antibiotic was indicated and a systemic antibiotic was prescribed
- Proportion of children who received a diagnosis for which a systemic antibiotic was not indicated and systemic antibiotic was prescribed

- Proportion of children who received first-line (or second-line) antibiotics according to recommendations for IMCI diagnoses for which specific antibiotic(s)are indicated

Only children with a diagnosis for which a systemic antibiotic was indicated will be counted in the denominator for the first proportion, only children with a diagnosis for which a systemic antibiotic was not indicated will be counted in the denominator of the second proportion, and only children who received an IMCI diagnosis requiring specific antibiotics will be counted in the denominator of the third proportion.

Diagnoses will be classified according to whether systemic antibiotics are indicated, based on IMCI and other relevant national guidelines as used for the CDSA, using the structured data entry and free text if feasible. Diagnosis classification will be defined in a separate document.

6.1.4 Antimalarial-related outcomes

Although the following outcomes need to share the same definition for a cross-country analysis, it is acknowledge that malaria testing strategies are different across countries. In Tanzania all febrile children (≥60 days) should be tested for malaria. In India febrile children should be tested only in high risk areas (or seasons), or in low risk areas if there is history of travel to a high risk area or when no other obvious cause of fever is detected. This difference will need be taken into account when reporting these outcomes. Untested febrile children will need to be described more in detail for India.

6.1.4.1 Proportion of febrile children tested for malaria at Day 0

A child will be considered to be febrile if, at Day 0, history of fever was reported by the caregiver before the consultation or temperature was recorded to be above or equal 37.5 C° or fever was recorded elsewhere in the registry (e.g. diagnosis).

A child will be considered tested for malaria if a malaria test was ordered during the consultation and a test result is available (i.e. positive, negative, invalid/indeterminate, reported but handwriting not readable).

Only febrile children will be counted in the denominator.

6.1.4.2 Proportion of malaria positive children prescribed an antimalarial

A malaria test will be considered positive if a malaria test was ordered during the consultation and was recorded as positive. If the test result is invalid/indeterminate, reported but handwriting not readable or not sure/not reported, the test will not be considered positive.

The child will be considered prescribed with an antimalarial if:

- One or more antimalarial drugs were selected from the antimalarial/treatment drop-down list
- Antimalarial drugs were not selected from the antimalarial/treatment drop-down list but antimalarial drugs were listed in the free text field (conditional to feasibility of assessing free text)

Only children with a positive malaria test result will be counted in the denominator.

6.1.4.3 Proportion of malaria negative children prescribed an antimalarial

A malaria test will be considered negative if a malaria test was ordered during the consultation and the result was negative. If the test result is invalid/indeterminate, or handwriting not readable or not sure/not reported, the test will not be considered negative.

Antimalarial drug prescription will be assessed as described in the point above.

Only children with a negative malaria test result will be counted in the denominator.

6.1.4.4 Proportion of untested children prescribed an antimalarial

A child will considered untested for malaria if, a malaria test was not ordered during the consultation or a malaria test was ordered during the consultation but a test result is not available.

Antimalarial drug prescription will be assessed as described in the point above.

Only children untested for malaria will be counted in the denominator.

6.1.5 Hypoxaemia-related outcomes – only in intervention arms

6.1.5.1 Proportion of children with severe, moderate and mild hypoxaemia, adjusted for sites at high altitude

Hypoxaemic children will be summarised disaggregated by groups based on the foillowing SpO2 values ranges: SpO2 < 90%, 90% ≤ SpO2 < 92% and 92% ≤ SpO2 < 94%. All children recruited will be in the denominator.

All facilities in India and Tanzania are in low altitude area and therefore no adjustment is required.

6.1.5.2 Proportion of children with hypoxaemia (according to differing cut-offs) with severe complication

Children with severe complication will be summarised disaggregated by groups based on the following SpO2 values ranges: SpO2 < 90%, 90% ≤ SpO2 < 92% and 92% ≤ SpO2 < 94%. spurious values and missing values. Each SpO2 group will be the denominator of each proportion.

6.1.5.3 Proportion of children with severe hypoxaemia not meeting any other clinical criteria for severe disease

Children with severe hypoxaemia (according to country's specific cut-off) not meeting any other clinical criteria for severe disease will be counted in the numerator. All children recruited will be in the denominator.

Severe disease will be defined in a separate document.

6.1.5.4 Proportion of children referred with hypoxaemia who receive oxygen at hospital

Children with hypoxaemia (according to country's specific cut-off) who received a referral advice at Day0 and oxygen at hospital (according to hospital records) will be counted in the numerator. All children recruited will be in the denominator.

Furthermore, children with hypoxaemia who were referred and children with hypoxaemia who were referred and admitted to hospital will be summarised using all children recruited as denominator.

Additionally, the proportions will be calculated and reported using only children with hypoxaemia as denominator.

6.1.6 Follow-up outcomes

6.1.6.1 Proportion of children attending scheduled follow-up at the same facility by Day 7

Attendance of scheduled follow-up at the same facility will be evaluated from:

- Scheduled and unscheduled visits form
  A child will be counted in the numerator if:

  - a follow-up visit advice is recorded at Day 0 and
  - a follow-up visit for the child is found in the scheduled/unscheduled visits form and
  - the follow-up visit is within 7 days of Day 0 (7th day included) and conducted at the enrollment facility

  All children recruited will be in the denominator.

  Additionally, the proportion will be calculated and reported using only children for whom a follow-up visit advice is recorded at Day0 as denominator.
  Children for whom a scheduled follow-up visit is not found in the scheduled/unscheduled visit form will be assumed to not have attended scheduled follow-up at the same facility by Day 7.

- Day 7 follow-up form
  A child will be counted in the numerator if:
  - a follow-up visit advice is recorded at Day 0 and
  - the caregiver reports visiting a government / public facility (non hospital) between child's enrollment and Day 7 follow-up visit date and
  - Day 7 follow-up date is within 7 days of Day 0, considering a + 3 days window (10th day included).

  All children recruited will be in the denominator.

  Additionally, the proportion will be calculated and reported using only children for whom a follow-up visit advice is recorded at Day0 as denominator.

Children for whom no Day7 data are available or withdrew prematurely from the study will be assumed to not have attended scheduled follow-up at the same facility / government or public facility by Day 7.

6.1.6.2 Proportion of children presenting for unscheduled follow-up to any health facility by Day 7

Attendance of unscheduled follow-up at any health facility will be evaluated from:

- Scheduled and unscheduled visits form
  A child will be counted in the numerator if:
  - a follow-up visit advice is not recorded at Day 0 and
  - a follow-up visit for the child is found in the scheduled/unscheduled visits form and
  - the follow-up visit is within 7 days of Day 0 (7th day included)

  All children recruited will be in the denominator.

  Additionally, the proportion will be calculated and reported using only children for whom a follow-up visit advice is not recorded at Day0 as denominator.

- Day 7 follow-up form
  A child will be counted in the numerator if:
  - a follow-up visit advice is not recorded at Day 0 and
  - the caregiver reports visiting a health facility (any, non hospital) between child's enrollment and Day 7 follow-up visit date and
  - Day 7 follow-up date is within 7 days of Day 0, considering a + 3 days window (10th day included).

  All children recruited will be in the denominator.

  Additionally, the proportion will be calculated and reported using only children for whom a follow-up visit advice is not recorded at Day0 as denominator.

Children for whom no Day7 data are available or withdrew prematurely from the study will be assumed to not have attended any health facility by Day 7.

6.2 Analysis Methods

6.2.1 Analytical Methods

Outcomes will be described by arm using summary statistics. In particular, counts and percentages will be used for binary variables and reported with one decimal place; mean and standard deviation (SD) or median (IQR) for continuous variables depending on their distribution. Mean and median will be displayed with one decimal place and SD and IQR will be presented with two decimal places.

The two primary outcomes will be assessed using a random effects logistic regression model with the cluster included as a random effect. If convergence issues arise due to sparse data, clusters may be dropped or combined to higher grouping level (e.g. district).

Results will be reported with odds ratios, risk differences and their associated 95% confidence intervals (CI). The control arm will be used as reference category.

Secondary outcomes and other binary outcomes of interest will be evaluated in the same way.

Other continuous outcomes of interest will be assessed using random effects linear regression model with the cluster included as a random effect. Results will be reported in terms of adjusted mean differences between arms with associated 95% CIs.

Modelling of secondary outcomes will be performed if numbers allow.

For interim analyses and DMC analyses, only the two primary outcomes will be presented. Modelling will not be performed and results will be reported in terms of summary statistics only.

6.2.2 Adjustment for Covariates

All models will be adjusted for stratification factors (facility type, location) and baseline variables that are found to be randomly imbalanced across arms. As described in Section 5.5, the imbalance will only be assessed visually and without performing any formal comparison.

6.2.3 Test of Assumptions, Actions to be taken

Continuous variables will be inspected using histograms: 1) to assess for outliers which may be queried for accuracy, and 2) to assess whether appropriate transformations are required for analyses.

As a multilevel logistic regression model is estimated using quadrature (rather likelihood methods), a quadrature check to test for adequate model fit will be performed. In case of unreliable model fit, generalised estimating equations will be used to fit the model which will provide population-averaged odds ratios and 95% confidence intervals.

6.2.4 Pre-planned Sensitivity Analyses

Sensitivity analyses on the primary outcomes are planned and will be conducted:

- On first encounters, which will include only the first enrolment episode of each child during the study period (as some children may attend the facility on more than one occasion). Separate presentations are identifiable from Day0 form as the caregiver is asked whether the child attended previously and within 28 days. This is the minimum number of days required for the disease episode to be considered a new one and not a repeat visit.
- Looking at different hospitalisation appropriateness (based on different definitions of primary and secondary hospitalisations). Same day (Day0), 3 days and 7 days from Day0 will be used as cut-offs for different primary and secondary hospitalisation definitions.
- Looking at different referral definitions:
    - A child will be considered to be referred if, at Day0, caregiver confirms that the child was urgently referred *and* the research assistant confirms that an urgent referral advice was recorded for the child[2].
    - A child will be considered to be referred if, at Day0, the research assistant confirms that an urgent referral advice was recorded for the child. If urgency is unknown, referral will be assumed to be urgent.

Additionally, a set of sensitivity analyses on the primary outcomes shall be carried out in case of substantial missing data (e.g. >10%) to evaluate the impact of each method on the primary outcomes results and take them into account when interpreting the final analysis results. Planned methods will include:

- Worst case scenario
- Complete cases
- Multiple imputations with chained equations [11, 12]

Details of the methods are provided in Section 6.3.

Sensitivity analyses will only be performed for the final statistical analysis and not for interim analyses and DMC reports.

6.2.5 Pre-specified Subgroups Analysis

Subgroup analysis of the primary outcomes are planned to assess effect modifiers, specifically:

- Age (under 2 months, 2 – 12 months, 13 – 59 months) – as recorded at Day0
- Sex (male, female) – as recorded at Day0
- Presentation with cough or difficulty breathing (yes, no) – as recorded at Day0
- Diagnosis (severe, non-severe)
- Referral (yes, no)
- Antimicrobial drug prescription (yes, no). Antimicrobials are defined as antimalarials and antibiotics, according to the same definition used for the outcomes.
- Stratification factors (location and facility type)

Effect modification will be assessed by incorporating an interaction between the time period and the potential effect modifier in the model, acknowledging that power will be low. Stratified model results will only be presented if the interaction term is significant, defined as p-value<0.2.

For interim analyses and DMC analyses, no subgroup analysis is planned.

[2] Urgency, as reported by the caregiver, was only collected in Tanzania. For India, urgent referrals will be defined based on the registry alone, as described for the primary analysis.

6.3 Missing Data

Missing values will be primarily due to children lost to follow-up and not reached at Day 7 and Day 28.

The primary analysis will be performed assuming the best case scenario (alive and not hospitalised) for children lost to follow-up or withdrawn.

As described in Section 6.2.4, a set of sensitivity analysis will be performed in case of substantial missing data on the primary outcomes (e.g. >10%). Missing data will be treated as outline below:

- Worst case scenario: children lost to follow-up or withdrawn will be assumed as dead or hospitalised.
- Complete cases: children lost to follow-up or withdrawn will be excluded.
- Multiple imputations with chained equations: under the assumption that children are lost to follow-up or withdrawn at random, chained equations with 50 imputed dataset, separately by treatment group, will be used. Relevant baseline characteristics and all available follow-up data will be used to impute death and hospitalisation (both primary and secondary) as binary variables.

Additionally, missing data will be described by arm and by relevant indicators, such as referral and severe disease to visually inspect for imbalances.

6.4 Additional analyses

Primary outcomes will be further presented by adherence and indication groups (e.g. children for which pulse oximetry was indicated and was used, children for which pulse oximetry was indicated but was not used, etc.).

6.5 Harms

Safety of the interventions will be evaluated in terms of deaths and secondary hospitalisations that occurred throughout the course of the study. Deaths, secondary hospitalisations and both combined will be reported as counts and percentages. Summary statistics will be presented separately for each intervention as outlined in Section 4.3.

6.6 Statistical Software

Statistical analysis will be performed using one of the standard statistical packages such as R v4.0.3 or higher versions [13]. The software and the version used for performing the analysis will be stated in the final statistical report.

7 REFERENCES

7.1 SOPs, study-specific Documents

A. TIMCI Data Management Plan.
B. TIMCI Data Monitoring Committee Charter.

7.2 External references

1. Gamble C, Krishan A, Stocken D, Lewis S, Juszczak E, Doré C, et al. Guidelines for the Content of Statistical Analysis Plans in Clinical Trials. JAMA. 2017 19;318(23):2337–43.

2. Costello, A., Dalglish, S. & on behalf of the Strategic Review Study Team. Towards a Grand Convergence for child survival and health: A strategic review of options for the future building on lessons learnt from IMNCI. (2016).

3. Wiens, B. L. A fixed sequence Bonferroni procedure for testing multiple endpoints. Pharmaceutical Statistics 2, 211–215 (2003).

4. Wiens, B. L. & Dmitrienko, A. The fallback procedure for evaluating a single family of hypotheses. J Biopharm Stat 15, 929–942 (2005).

5. Alosh, M., Bretz, F. & Huque, M. Advanced multiplicity adjustment methods in clinical trials. *Statistics in Medicine* **33**, 693–713 (2014).

6. Diaz T, Strong KL, Cao B, Guthold R, Moran AC, Moller AB, et al. A call for standardised age-disaggregated health data. The Lancet Healthy Longevity [Internet]. 2021 Jul;2(7):e436–43. Available from: https://linkinghub.elsevier.com/retrieve/pii/S266675682100115X

7. Geneva: World Health Organization; 2018. Licence: CC BY-NC-SA 3.0 IGO.

8. https://www.who.int/publications/i/item/2021-aware-classification

9. Pauwels et al, Hospital antibiotic prescribing patterns in adult patients according to the WHO Access, Watch and Reserve classification (AWaRe): results from a worldwide point prevalence survey in 69 countries, *Journal of Antimicrobial Chemotherapy*, Volume 76, Issue 6, June 2021, Pages 1614–1624.

10. The CONSORT Group. CONSORT Statement [Internet]. [cited 2012 Mar 22]. Available from: http://www.consort-statement.org/consort-statement/13-19---results/item15_baseline-data/.

11. Jakobsen JC, Gluud C, Wetterslev J, Winkel P. When and how should multiple imputation be used for handling missing data in randomised clinical trials – a practical guide with flowcharts. BMC Med Res Methodol. 2017 Dec 6;17(1):162.

12. Sullivan TR, White IR, Salter AB, Ryan P, Lee KJ. Should multiple imputation be the method of choice for handling missing data in randomized trials? Stat Methods Med Res. 2016 Dec 19;0962280216683570.

13. R Core Team (2020). R: A language and environment for statistical computing. R Foundation for Statistical Computing, Vienna, Austria. URL https://www.R-project.org/.

8 APPENDIX A

The age of the child is expressed differently for *young infants* (i.e. children up to 59 days) and for children between 60 days and 59 months (sometimes more simply referred to as "*2-59 months*")

- It is grouped in three categories ([1-6 days][3], [7-27 days], [28-59 days]) for young infants;
- It is expressed in months for children from 60 days to 59 months.

Figure 1) Recommended standardised age disaggregation groups for data analysis by life stage [6].

	Recommended age grouping	Disease burden and health risk*	Examples of key prevention and health promotion interventions	Living conditions and societal factors
Early neonates	0–6 days	A third of all neonatal deaths occur on the day of birth, and nearly 75% occur within the first week of life	Immediate breastfeeding; vaccination (eg, for BCG and hepatitis B) and screening for genetic, endocrine, and metabolic disorders at birth or within the first 24 h of life	Early neonates might be in facility care immediately after birth, especially if premature and low-weight or ill, but are most commonly cared for at home by family
Late neonates	7–27 days	The first 27 days of life (neonatal or newborn period) are the most crucial for survival; neonates accounted for 2·5 million deaths (47% of all deaths under the age of 5 years) in 2019;[31] causes of death in this age group differ from those in early neonatal and post-neonatal infants and a large proportion is due to congenital anomalies	Ensure neonates have received vaccines at birth, check weight, assess for birth defects, and promote the continuation of exclusive breastfeeding	Commonly cared for at home by family or caretakers
Post-neonatal infants	28–364 days	The first year of life after the neonatal phase is the second riskiest period for child survival; about 24% of all deaths under the age of 5, in 2019, occurred in this age group,[31] with pneumonia, diarrhoea, and malaria as leading causes of death	Completion of common vaccines schedule (diphtheria, tetanus, pertussis, *Haemophilus influenzae* type b, hepatitis B, poliovirus, pneumococcal, rotavirus, measles-rubella, and two doses of seasonal influenza);[32] continuation of breastfeeding with weaning and introduction of complementary foods when appropriate; long-lasting insecticidal nets and intermittent preventive treatment for infants in malaria-endemic countries	Commonly cared for at home by family, but can begin to go to day care
Young children	1–4 years	This group has the greatest reductions in mortality of all age groups to date, but mortality in this group remains fairly high in many countries; environmental exposures during the first 3 years of life can affect a child's developmental trajectory and lead to an increased risk of physical and psychological illness, affecting health and wellbeing in later life[27–29]	Diphtheria, tetanus, pertussis, and measles-rubella boosters and seasonal influenza vaccine;[32] completion of weaning; long-lasting insecticidal nets and seasonal malaria chemoprevention in malaria endemic countries; early childhood programmes; environmental and policy interventions (eg, clean water and sanitation, fluoridation, safe playgrounds, and appropriate car seats)	Children can begin to attend preschool or day care and often play with other children

The age of the child is available in the TIMCI database with different levels of accuracy depending on the information primarily shared by the caregiver. It can be either calculated as number of days between date of consultation and date of birth or estimated by categories.

Calculated Age

The date of creation of the ODK form is considered to be the date of consultation. The date of birth can be entered with three different levels of accuracy:

- **DD/MM/YYYY**
  If the exact date of birth is known, the age in month is derived from the age in days through a division by 30.44 $age_{\text{month}} = \frac{age_{\text{days}}}{30.44}$
  The age is converted to the corresponding categories for young infants to keep the age granularity necessary while avoiding being able to indirectly recalculate the date of birth from the date of consultation and age in days:

$$\begin{cases} 1 \leq if\ age_{\text{days}} < 7 & [1-6\text{d}] \\ 7 \leq if\ age_{\text{days}} < 28 & [7-27\text{d}] \\ 28 \leq if\ age_{\text{days}} < 60 & [28-59\text{d}] \end{cases}$$

- **MM/YYYY and YYYY**

[3] The first day of life (or day of birth) is an exclusion criterion for TIMCI.

When the date of birth is not exactly known with a day accuracy, the age can only be known within a range of maximum and minimum ages.
The following approach is used:

- Young infants: those children who have their minimal age (variable *a3-a3_a_6a* in the Day 0 ODK form) in the interval [1-59 days]
- Children from 60 days to 59 months: those children who have their minimal age (variable *a3-a3_a_6a* in the Day 0 ODK form) in the interval [60 days - 59 months]

When only the year of birth is known, it is attempted to reduce the possible age range by clarifying the season of the birth of the child:

- January - March
- April - June
- July - September
- October – December
- Unknown (code: 98), the age range remains the one determined by the year of birth and the date of consultation

Estimated age

If the caregiver is not able to provide any elements of the date of birth, an available alternative is to select the category corresponding to the estimated age of the child.

The available categories that are defined in the data collection tool are as follow:

- Under 1 year
  - Under 1 month
    - Less than 1 day (first day of life) ⇒ children excluded from the study
    - 1-6 days
    - 7-28 days
  - 1 month
  - 2 months
  - 3 months
  - 4 months
  - 5 months
  - 6 months
  - 7 months
  - 8 months
  - 9 months
  - 10 months
  - 11 months
- From 1 to under 2 years
  - 1 year and 0-2 months (12-14 months)
  - 1 year and 3-5 months (15-17 months)
  - 1 year and 6-8 months (18-20 months)
  - 1 year and 9-11 months (21-23 months)
- From 2 to under 3 years
- From 3 to under 4 years
- From 4 to under 5 years
