# Supplementary material for: Effectiveness of introducing pulse oximetry and clinical decision support algorithms for the management of sick children in primary care in India and Tanzania on hospitalisation and mortality: the TIMCI pragmatic cluster randomised controlled trial
Source: eClinicalMedicine. 2025 Jul 3;85:103306. doi: 10.1016/j.eclinm.2025.103306 (PMC12271772; doi:10.1016/j.eclinm.2025.103306)
Supplement: 01_RCT_S1 [file mmc5.docx]

## Supplementary file S1 – Characteristics of facilities and recruitment by facilities

### Characteristics of recruiting facilities

| Variables | Statistics/Categories | Control (n=74) | PO (n=74) |
| --- | --- | --- | --- |
| District | Deoria | 24.3% (18) | 25.7% (19) |
|  | Kaliua | 12.2% (9) | 6.8% (5) |
|  | Sengerema | 6.8% (5) | 10.8% (8) |
|  | Sitapur | 23.0% (17) | 21.6% (16) |
|  | Tanga | 9.5% (7) | 10.8% (8) |
|  | Unnao | 24.3% (18) | 24.3% (18) |
| Location | Rural | 93.2% (69) | 93.2% (69) |
|  | Urban | 6.8% (5) | 6.8% (5) |
| Type | PHC/Dispensary | 66.2% (49) | 66.2% (49) |
|  | CHC/Health center | 33.8% (25) | 33.8% (25) |
| Number children per PHC/Dispensary | Median, min-max | 260, 18-2471 | 322, 35-2043 |
| Number children per CHC/Health center | Median, min-max | 1011, 169-3883 | 1000, 120-4741 |

### Distribution of children by characteristics of recruiting facilities

| Variables | Statistics/Categories | Control (n=57506) | PO (n=60980) |
| --- | --- | --- | --- |
| District | Deoria | 13.0% (7481) | 12.3% (7500) |
|  | Kaliua | 29.0% (16676) | 14.4% (8777) |
|  | Sengerema | 8.1% (4646) | 16.6% (10136) |
|  | Sitapur | 16.5% (9460) | 15.7% (9553) |
|  | Tanga | 21.1% (12119) | 28.0% (17101) |
|  | Unnao | 12.4% (7124) | 13.0% (7913) |
| Location | Rural | 79.5% (45714) | 80.2% (48877) |
|  | Urban | 20.5% (11792) | 19.8% (12103) |
| Type | PHC/Dispensary | 50.2% (28873) | 46.7% (28500) |
|  | CHC/Health center | 49.8% (28633) | 53.3% (32480) |

### Characteristics of recruiting facilities - India

| Variables | Statistics/Categories | Control (n=53) | PO (n=53) |
| --- | --- | --- | --- |
| District | Deoria | 34.0% (18) | 35.8% (19) |
|  | Sitapur | 32.1% (17) | 30.2% (16) |
|  | Unnao | 34.0% (18) | 34.0% (18) |
| Type | CHC | 39.6% (21) | 37.7% (20) |
|  | PHC | 60.4% (32) | 62.3% (33) |
| Location | Rural | 100.0% (53) | 100.0% (53) |
| Number children per PHC | Median, min-max | 209.5, 18-390 | 228, 35-777 |
| Number children per CHC | Median, min-max | 819, 169-1200 | 955, 120-1383 |

### Distribution of children by characteristics of recruiting facilities - India

| Variables | Statistics/Categories | Control (n=24065) | PO (n=24966) |
| --- | --- | --- | --- |
| District | Deoria | 31.1% (7481) | 30.0% (7500) |
|  | Sitapur | 39.3% (9460) | 38.3% (9553) |
|  | Unnao | 29.6% (7124) | 31.7% (7913) |
| Location | Rural | 100.0% (24065) | 100.0% (24966) |
| Type | CHC | 72.5% (17455) | 66.5% (16600) |
|  | PHC | 27.5% (6610) | 33.5% (8366) |

### Characteristics of recruiting facilities - Tanzania

| Variables | Statistics/Categories | Control (n=21) | PO (n=21) | PO+CDSA (n=24) |
| --- | --- | --- | --- | --- |
| District | Kaliua | 42.9% (9) | 23.8% (5) | 29.2% (7) |
|  | Sengerema | 23.8% (5) | 38.1% (8) | 41.7% (10) |
|  | Tanga | 33.3% (7) | 38.1% (8) | 29.2% (7) |
| Type | dispensary | 81.0% (17) | 76.2% (16) | 75.0% (18) |
|  | health center | 19.0% (4) | 23.8% (5) | 25.0% (6) |
| Location | Rural | 76.2% (16) | 76.2% (16) | 75.0% (18) |
|  | Urban | 23.8% (5) | 23.8% (5) | 25.0% (6) |
| Number children per dispensary | Median, min-max | 1233, 520-2471 | 1185.5, 514-2043 | 1442, 456-3169 |
| Number children per health center | Median, min-max | 2758, 1779-3883 | 3042, 2059-4741 | 2314, 1034-3070 |

### Distribution of children by characteristics of recruiting facilities - Tanzania

| Variables | Statistics/Categories | Control (n=33441) | PO (n=36014) | PO+CDSA (n=39191) |
| --- | --- | --- | --- | --- |
| District | Kaliua | 49.9% (16676) | 24.4% (8777) | 31.3% (12284) |
|  | Sengerema | 13.9% (4646) | 28.1% (10136) | 38.1% (14913) |
|  | Tanga | 36.2% (12119) | 47.5% (17101) | 30.6% (11994) |
| Location | Rural | 64.7% (21649) | 66.4% (23911) | 65.2% (25558) |
|  | Urban | 35.3% (11792) | 33.6% (12103) | 34.8% (13633) |
| Type | dispensary | 66.6% (22263) | 55.9% (20134) | 66.3% (25990) |
|  | health center | 33.4% (11178) | 44.1% (15880) | 33.7% (13201) |
