# Supplementary material for: Effectiveness of introducing pulse oximetry and clinical decision support algorithms for the management of sick children in primary care in India and Tanzania on hospitalisation and mortality: the TIMCI pragmatic cluster randomised controlled trial
Source: eClinicalMedicine. 2025 Jul 3;85:103306. doi: 10.1016/j.eclinm.2025.103306 (PMC12271772; doi:10.1016/j.eclinm.2025.103306)
Supplement: 01_RCT_S2 [file mmc6.docx]

## Supplementary file S2 – Summary of Day 0 indicators by arms and lost to follow-up status

Missing values are due predominantly to children lost to follow-up and not reached at Day7 and Day28.

The tables below describes relevant indicators recorded at Day0 by follow-up status.

### Indicators summary by arms and lost to follow-up status - Day 7

|  | Lost to follow-up at Day 7 | | | Successful follow-up at Day 7 | | |
| --- | --- | --- | --- | --- | --- | --- |
|  | Control | PO | PO+CDSA | Control | PO | PO+CDSA |
| Urgent referral |  |  |  |  |  |  |
| Combined | 0.4% (23/5829) | 0.9% (44/4877) | - | 0.3% (131/51677) | 0.6% (362/56103) | - |
| India | 0.0% (0/3723) | 0.3% (11/3276) | - | 0.1% (19/20342) | 0.4% (84/21690) | - |
| Tanzania | 1.1% (23/2106) | 2.1% (33/1601) | 1.4% (35/2491) | 0.4% (112/31335) | 0.8% (278/34413) | 1.8% (667/36700) |
| Severe diagnosis without hypoxaemia |  |  |  |  |  |  |
| Combined | 2.2% (127/5829) | 1.6% (78/4877) | - | 1.6% (830/51677) | 2.2% (1231/56103) | - |
| India | 0.6% (21/3723) | 0.3% (9/3276) | - | 0.8% (172/20342) | 0.6% (129/21690) | - |
| Tanzania | 5.0% (106/2106) | 4.3% (69/1601) | 4.7% (118/2491) | 2.1% (658/31335) | 3.2% (1102/34413) | 3.3% (1194/36700) |
| Severe diagnosis with hypoxaemia |  |  |  |  |  |  |
| Combined | 0.0% (0/5829) | 0.3% (16/4877) | - | 0.0% (0/51677) | 0.3% (174/56103) | - |
| India | 0.0% (0/3723) | 0.2% (8/3276) | - | 0.0% (0/20342) | 0.4% (82/21690) | - |
| Tanzania | 0.0% (0/2106) | 0.5% (8/1601) | 0.5% (12/2491) | 0.0% (0/31335) | 0.3% (92/34413) | 0.3% (93/36700) |

### Indicators summary by arms and lost to follow-up status - Day 28

|  | Lost to follow-up at Day 28 | | | Successful follow-up at Day 28 | | |
| --- | --- | --- | --- | --- | --- | --- |
|  | Control | PO | PO+CDSA | Control | PO | PO+CDSA |
| Urgent referral |  |  |  |  |  |  |
| Combined | 0.3% (15/5552) | 0.8% (37/4570) | - | 0.3% (139/51954) | 0.7% (369/56410) | - |
| India | 0.0% (1/3857) | 0.3% (12/3434) | - | 0.1% (18/20208) | 0.4% (83/21532) | - |
| Tanzania | 0.8% (14/1695) | 2.2% (25/1136) | 0.9% (14/1638) | 0.4% (121/31746) | 0.8% (286/34878) | 1.8% (688/37553) |
| Severe diagnosis without hypoxaemia |  |  |  |  |  |  |
| Combined | 2.1% (114/5552) | 1.5% (67/4570) | - | 1.6% (843/51954) | 2.2% (1242/56410) | - |
| India | 0.6% (25/3857) | 0.4% (15/3434) | - | 0.8% (168/20208) | 0.6% (123/21532) | - |
| Tanzania | 5.3% (89/1695) | 4.6% (52/1136) | 4.9% (81/1638) | 2.1% (675/31746) | 3.2% (1119/34878) | 3.3% (1231/37553) |
| Severe diagnosis with hypoxaemia |  |  |  |  |  |  |
| Combined | 0.0% (0/5552) | 0.4% (17/4570) | - | 0.0% (0/51954) | 0.3% (173/56410) | - |
| India | 0.0% (0/3857) | 0.3% (11/3434) | - | 0.0% (0/20208) | 0.4% (79/21532) | - |
| Tanzania | 0.0% (0/1695) | 0.5% (6/1136) | 0.1% (2/1638) | 0.0% (0/31746) | 0.3% (94/34878) | 0.3% (103/37553) |

### Indicators summary by arms and lost to follow-up status - both Day 7 and Day 28

|  | Lost to follow-up at both FU | | | At least one successful follow-up | | |
| --- | --- | --- | --- | --- | --- | --- |
|  | Control | PO | PO+CDSA | Control | PO | PO+CDSA |
| Urgent referral |  |  |  |  |  |  |
| Combined | 0.3% (12/3619) | 0.8% (23/2843) | - | 0.3% (142/53887) | 0.7% (383/58137) | - |
| India | 0.0% (0/2612) | 0.4% (8/2203) | - | 0.1% (19/21453) | 0.4% (87/22763) | - |
| Tanzania | 1.2% (12/1007) | 2.3% (15/640) | 0.5% (6/1116) | 0.4% (123/32434) | 0.8% (296/35374) | 1.8% (696/38075) |
| Severe diagnosis without hypoxaemia |  |  |  |  |  |  |
| Combined | 2.0% (72/3619) | 1.2% (35/2843) | - | 1.6% (885/53887) | 2.2% (1274/58137) | - |
| India | 0.5% (12/2612) | 0.4% (8/2203) | - | 0.8% (181/21453) | 0.6% (130/22763) | - |
| Tanzania | 6.0% (60/1007) | 4.2% (27/640) | 4.3% (48/1116) | 2.2% (704/32434) | 3.2% (1144/35374) | 3.3% (1264/38075) |
| Severe diagnosis with hypoxaemia |  |  |  |  |  |  |
| Combined | 0.0% (0/3619) | 0.3% (9/2843) | - | 0.0% (0/53887) | 0.3% (181/58137) | - |
| India | 0.0% (0/2612) | 0.3% (7/2203) | - | 0.0% (0/21453) | 0.4% (83/22763) | - |
| Tanzania | 0.0% (0/1007) | 0.3% (2/640) | 0.1% (1/1116) | 0.0% (0/32434) | 0.3% (98/35374) | 0.3% (104/38075) |
