# Supplementary material for: Effectiveness of introducing pulse oximetry and clinical decision support algorithms for the management of sick children in primary care in India and Tanzania on hospitalisation and mortality: the TIMCI pragmatic cluster randomised controlled trial
Source: eClinicalMedicine. 2025 Jul 3;85:103306. doi: 10.1016/j.eclinm.2025.103306 (PMC12271772; doi:10.1016/j.eclinm.2025.103306)
Supplement: 01_RCT_S3 [file mmc7.docx]

## Supplementary file S3 – Summary of severe complications

### Proportion of children who experienced a severe complication at any point during the study duration

The table below summarises severe complications (death or secondary hospitalisation) that occurred throughout the duration of the study.

### Deaths and secondary hospitalisations summary - infants under 2 months of age

|  | Control | PO | PO+CDSA |
| --- | --- | --- | --- |
| Deaths |  |  |  |
| Combined | 0.3% (10/3188) | 0.7% (27/4012) | - |
| India | 0.3% (4/1176) | 1.1% (17/1582) | - |
| Tanzania | 0.3% (6/2012) | 0.4% (10/2430) | 0.5% (13/2386) |
| Hospitalisations without referral |  |  |  |
| Combined | 0.3% (11/3188) | 0.4% (16/4012) | - |
| India | 0.3% (4/1176) | 0.5% (8/1582) | - |
| Tanzania | 0.3% (7/2012) | 0.3% (8/2430) | 0.9% (21/2386) |
| Hospitalisations with referral but delayed |  |  |  |
| Combined | 0.0% (1/3188) | 0.1% (6/4012) | - |
| India | 0.0% (0/1176) | 0.1% (1/1582) | - |
| Tanzania | 0.0% (1/2012) | 0.2% (5/2430) | 0.0% (1/2386) |
| Severe complications |  |  |  |
| Combined | 0.7% (22/3188) | 1.1% (46/4012) | - |
| India | 0.7% (8/1176) | 1.6% (25/1582) | - |
| Tanzania | 0.7% (14/2012) | 0.9% (21/2430) | 1.4% (34/2386) |

### Deaths and secondary hospitalisations summary - children 2-59 months of age

|  | Control | PO | PO+CDSA |
| --- | --- | --- | --- |
| Deaths |  |  |  |
| Combined | 0.0% (25/54318) | 0.1% (52/56968) | - |
| India | 0.0% (10/22889) | 0.1% (20/23384) | - |
| Tanzania | 0.0% (15/31429) | 0.1% (32/33584) | 0.1% (45/36805) |
| Hospitalisations without referral |  |  |  |
| Combined | 0.2% (109/54318) | 0.3% (173/56968) | - |
| India | 0.1% (29/22889) | 0.2% (50/23384) | - |
| Tanzania | 0.3% (80/31429) | 0.4% (123/33584) | 0.4% (161/36805) |
| Hospitalisations with referral but delayed |  |  |  |
| Combined | 0.0% (5/54318) | 0.0% (5/56968) | - |
| India | 0.0% (0/22889) | 0.0% (1/23384) | - |
| Tanzania | 0.0% (5/31429) | 0.0% (4/33584) | 0.0% (11/36805) |
| Severe complications |  |  |  |
| Combined | 0.3% (136/54318) | 0.4% (223/56968) | - |
| India | 0.2% (37/22889) | 0.3% (69/23384) | - |
| Tanzania | 0.3% (99/31429) | 0.5% (154/33584) | 0.6% (206/36805) |
