# Supplementary material for: Effectiveness of introducing pulse oximetry and clinical decision support algorithms for the management of sick children in primary care in India and Tanzania on hospitalisation and mortality: the TIMCI pragmatic cluster randomised controlled trial
Source: eClinicalMedicine. 2025 Jul 3;85:103306. doi: 10.1016/j.eclinm.2025.103306 (PMC12271772; doi:10.1016/j.eclinm.2025.103306)
Supplement: 01_RCT_S4 [file mmc8.docx]

## Supplementary file S4 - Summary of primary outcomes results

The primary outcomes are assessed using generalised estimating equation for logistic regression, with facilities as clusters. Estimates for the intervention effect on the outcomes are shown in terms of odds ratios and risk difference (with the control arm being the reference category) and their associated 95% confidence intervals. Results from both univariate and multivariate models are presented when numbers allowed. Not estimable intervention effects are denoted in the summary table below as “NE”.

Multivariate models are adjusted for districts, facility type (dispensaries or PHCs/health centers or CHCs) and previous care or treatment at time of presenting at the facility (no/yes/unknown).

### Summary of primary outcomes results - infants under 2 months of age

| Outcome | Analysis | N (%) Control | N (%) PO | N (%) PO+CDSA | Unadjusted | p-value | Adjusted | p-value | |
| --- | --- | --- | --- | --- | --- | --- | --- | --- | --- |
| Severe complications by Day7 | | | | | | | | |  |
| Primary analysis ITT | Combined | 16 (0.5%) | 27 (0.7%) | - | 1.371 (0.715, 2.631) 0.2% (-0.2%, 0.5%) | 0.343 | NE | - | |
|  | India | 8 (0.7%) | 15 (0.9%) | - | 1.404 (0.579, 3.406) 0.3% (-0.4%, 0.9%) | 0.452 | NE | - | |
|  | Tanzania | 8 (0.4%) | 12 (0.5%) | - | 1.164 (0.472, 2.869) 0.1% (-0.4%, 0.5%) | 0.741 | NE | - | |
|  |  | 8 (0.4%) | - | 21 (0.9%) | 2.108 (0.875, 5.080) 0.5% (-0.1%, 1.0%) | 0.097 | NE | - | |
| Primary analysis CC | Combined | 16 (0.5%) | 27 (0.7%) | - | 1.363 (0.710, 2.617) 0.2% (-0.2%, 0.6%) | 0.352 | NE | - | |
|  | India | 8 (0.7%) | 15 (1%) | - | 1.383 (0.574, 3.336) 0.3% (-0.5%, 1.0%) | 0.470 | NE | - | |
|  | Tanzania | 8 (0.4%) | 12 (0.5%) | - | 1.165 (0.473, 2.872) 0.1% (-0.4%, 0.5%) | 0.739 | NE | - | |
|  |  | 8 (0.4%) | - | 21 (0.9%) | 2.140 (0.886, 5.170) 0.5% (-0.1%, 1.1%) | 0.091 | NE | - | |
| Sensitivity analysis-first encounters | Combined | 16 (0.5%) | 26 (0.7%) | - | 1.367 (0.701, 2.665) 0.2% (-0.2%, 0.6%) | 0.359 | NE | - | |
|  | India | 8 (0.7%) | 15 (1%) | - | 1.400 (0.578, 3.391) 0.3% (-0.4%, 0.9%) | 0.456 | NE | - | |
|  | Tanzania | 8 (0.4%) | 11 (0.5%) | - | 1.128 (0.432, 2.943) 0.1% (-0.4%, 0.5%) | 0.806 | NE | - | |
|  |  | 8 (0.4%) | - | 21 (0.9%) | 2.184 (0.906, 5.262) 0.5% (-0.1%, 1.1%) | 0.082 | NE | - | |
| Sensitivity analysis-hosp cut-off Day0 | Combined | 16 (0.5%) | 27 (0.7%) | - | 1.371 (0.715, 2.631) 0.2% (-0.2%, 0.5%) | 0.343 | NE | - | |
|  | India | 8 (0.7%) | 15 (0.9%) | - | 1.404 (0.579, 3.406) 0.3% (-0.4%, 0.9%) | 0.452 | NE | - | |
|  | Tanzania | 8 (0.4%) | 12 (0.5%) | - | 1.254 (0.506, 3.111) 0.1% (-0.4%, 0.6%) | 0.625 | NE | - | |
|  |  | 8 (0.4%) | - | 23 (1%) | 2.458 (0.997, 6.060) 0.6% (-0.1%, 1.2%) | 0.051 | NE | - | |
| Sensitivity analysis-hosp cut-off Day3 | Combined | 16 (0.5%) | 24 (0.6%) | - | 1.207 (0.624, 2.334) 0.1% (-0.2%, 0.5%) | 0.577 | NE | - | |
|  | India | 8 (0.7%) | 14 (0.9%) | - | 1.312 (0.532, 3.233) 0.2% (-0.5%, 0.9%) | 0.556 | NE | - | |
|  | Tanzania | 8 (0.4%) | 10 (0.4%) | - | 0.829 (0.359, 1.913) -0.1% (-0.6%, 0.4%) | 0.660 | NE | - | |
|  |  | 8 (0.4%) | - | 20 (0.8%) | 1.742 (0.737, 4.119) 0.4% (-0.2%, 0.9%) | 0.206 | NE | - | |
| Sensitivity analysis-hosp cut-off Day7 | Combined | 15 (0.5%) | 24 (0.6%) | - | 1.260 (0.666, 2.385) 0.1% (-0.2%, 0.5%) | 0.478 | NE | - | |
|  | India | 8 (0.7%) | 14 (0.9%) | - | 1.312 (0.532, 3.233) 0.2% (-0.5%, 0.9%) | 0.556 | NE | - | |
|  | Tanzania | 7 (0.3%) | 10 (0.4%) | - | 0.913 (0.456, 1.829) -0.0% (-0.4%, 0.3%) | 0.798 | NE | - | |
|  |  | 7 (0.3%) | - | 20 (0.8%) | 1.930 (0.918, 4.058) 0.4% (-0.1%, 0.9%) | 0.083 | NE | - | |
| Sensitivity analysis-referral caregiver and registry | Combined | - | - | - | - | - | - | - | |
|  | India | - | - | - | - | - | - | - | |
|  | Tanzania | 8 (0.4%) | 10 (0.4%) | - | 0.883 (0.375, 2.081) -0.1% (-0.5%, 0.4%) | 0.776 | NE | - | |
|  |  | 8 (0.4%) | - | 22 (0.9%) | 1.997 (0.838, 4.760) 0.5% (-0.1%, 1.0%) | 0.118 | NE | - | |
| Sensitivity analysis-referral only registry | Combined | 15 (0.5%) | 24 (0.6%) | - | 1.260 (0.666, 2.385) 0.1% (-0.2%, 0.5%) | 0.478 | NE | - | |
|  | India | 8 (0.7%) | 14 (0.9%) | - | 1.312 (0.532, 3.233) 0.2% (-0.5%, 0.9%) | 0.556 | NE | - | |
|  | Tanzania | 7 (0.3%) | 10 (0.4%) | - | 0.944 (0.465, 1.917) -0.0% (-0.4%, 0.3%) | 0.873 | NE | - | |
|  |  | 7 (0.3%) | - | 21 (0.9%) | 2.109 (0.977, 4.552) 0.5% (-0.0%, 1.0%) | 0.057 | NE | - | |
| Appropriate referrals | | | | | | | | |  |
| Primary analysis ITT | Combined | 0 (0%) | 9 (0.2%) | - | NE | - | NE | - | |
|  | India | 0 (0%) | 5 (0.3%) | - | NE | - | NE | - | |
|  | Tanzania | 0 (0%) | 4 (0.2%) | - | NE | - | NE | - | |
|  |  | 0 (0%) | - | 6 (0.3%) | NE | - | NE | - | |
| Primary analysis CC | Combined | 0 (0%) | 9 (0.2%) | - | NE | - | NE | - | |
|  | India | 0 (0%) | 5 (0.3%) | - | NE | - | NE | - | |
|  | Tanzania | 0 (0%) | 4 (0.2%) | - | NE | - | NE | - | |
|  |  | 0 (0%) | - | 6 (0.3%) | NE | - | NE | - | |

### Summary of primary outcomes results - children 2-59 months of age

| Outcome | Analysis | N (%) Control | N (%) PO | N (%) PO+CDSA | Univariate | p-value | Multivariate | p-value |
| --- | --- | --- | --- | --- | --- | --- | --- | --- |
| Severe complications by Day7 | | | | | | | | |
| Primary analysis ITT | Combined | 77 (0.1%) | 143 (0.3%) | - | 1.770 (1.168, 2.683) 0.1% (0.0%, 0.2%) | 0.007 | 1.655 (1.115, 2.455) 0.1% (0.0%, 0.2%) | 0.012 |
|  | India | 25 (0.1%) | 48 (0.2%) | - | 1.819 (1.013, 3.266) 0.1% (0.0%, 0.2%) | 0.045 | NE | - |
|  | Tanzania | 52 (0.2%) | 95 (0.3%) | - | 1.732 (0.973, 3.083) 0.1% (-0.0%, 0.3%) | 0.062 | 1.460 (0.883, 2.414) 0.1% (-0.1%, 0.3%) | 0.140 |
|  |  | 52 (0.2%) | - | 128 (0.3%) | 2.130 (1.176, 3.858) 0.2% (0.0%, 0.3%) | 0.013 | 1.595 (0.957, 2.659) 0.1% (-0.0%, 0.3%) | 0.073 |
| Primary analysis CC | Combined | 77 (0.2%) | 143 (0.3%) | - | 1.752 (1.160, 2.645) 0.1% (0.0%, 0.2%) | 0.008 | 1.644 (1.109, 2.438) 0.1% (0.0%, 0.2%) | 0.013 |
|  | India | 25 (0.1%) | 48 (0.2%) | - | 1.790 (1.002, 3.200) 0.1% (-0.0%, 0.2%) | 0.049 | NE | - |
|  | Tanzania | 52 (0.2%) | 95 (0.3%) | - | 1.717 (0.965, 3.055) 0.1% (-0.0%, 0.3%) | 0.066 | 1.470 (0.889, 2.432) 0.1% (-0.1%, 0.3%) | 0.134 |
|  |  | 52 (0.2%) | - | 128 (0.4%) | 2.125 (1.176, 3.840) 0.2% (0.0%, 0.3%) | 0.013 | 1.612 (0.965, 2.695) 0.1% (-0.0%, 0.3%) | 0.068 |
| Sensitivity analysis-first encounters | Combined | 62 (0.1%) | 123 (0.2%) | - | 1.913 (1.230, 2.977) 0.1% (0.0%, 0.2%) | 0.004 | 1.799 (1.186, 2.730) 0.1% (0.0%, 0.2%) | 0.006 |
|  | India | 24 (0.1%) | 47 (0.2%) | - | 1.856 (1.023, 3.369) 0.1% (0.0%, 0.2%) | 0.042 | NE | - |
|  | Tanzania | 38 (0.1%) | 76 (0.3%) | - | 1.989 (1.039, 3.809) 0.1% (-0.0%, 0.3%) | 0.038 | 1.708 (0.989, 2.947) 0.1% (-0.0%, 0.3%) | 0.055 |
|  |  | 38 (0.1%) | - | 103 (0.3%) | 2.312 (1.188, 4.499) 0.2% (0.0%, 0.3%) | 0.014 | 1.774 (1.012, 3.111) 0.2% (-0.0%, 0.3%) | 0.045 |
| Sensitivity analysis-hosp cut-off Day0 | Combined | 79 (0.1%) | 146 (0.3%) | - | 1.762 (1.169, 2.656) 0.1% (0.0%, 0.2%) | 0.007 | 1.657 (1.127, 2.434) 0.1% (0.0%, 0.2%) | 0.010 |
|  | India | 25 (0.1%) | 49 (0.2%) | - | 1.853 (1.035, 3.317) 0.1% (0.0%, 0.2%) | 0.038 | NE | - |
|  | Tanzania | 54 (0.2%) | 97 (0.3%) | - | 1.686 (0.960, 2.962) 0.1% (-0.0%, 0.3%) | 0.069 | 1.434 (0.882, 2.331) 0.1% (-0.1%, 0.3%) | 0.146 |
|  |  | 54 (0.2%) | - | 133 (0.4%) | 2.116 (1.183, 3.785) 0.2% (0.0%, 0.4%) | 0.012 | 1.576 (0.965, 2.572) 0.1% (-0.0%, 0.3%) | 0.069 |
| Sensitivity analysis-hosp cut-off Day3 | Combined | 77 (0.1%) | 141 (0.2%) | - | 1.736 (1.142, 2.641) 0.1% (0.0%, 0.2%) | 0.010 | 1.621 (1.087, 2.418) 0.1% (0.0%, 0.2%) | 0.018 |
|  | India | 25 (0.1%) | 47 (0.2%) | - | 1.784 (0.989, 3.219) 0.1% (-0.0%, 0.2%) | 0.055 | NE | - |
|  | Tanzania | 52 (0.2%) | 94 (0.3%) | - | 1.696 (0.947, 3.036) 0.1% (-0.0%, 0.2%) | 0.075 | 1.438 (0.867, 2.383) 0.1% (-0.1%, 0.3%) | 0.159 |
|  |  | 52 (0.2%) | - | 126 (0.3%) | 2.098 (1.161, 3.793) 0.2% (0.0%, 0.3%) | 0.014 | 1.569 (0.941, 2.618) 0.1% (-0.0%, 0.3%) | 0.084 |
| Sensitivity analysis-hosp cut-off Day7 | Combined | 76 (0.1%) | 141 (0.2%) | - | 1.750 (1.154, 2.656) 0.1% (0.0%, 0.2%) | 0.008 | 1.632 (1.097, 2.427) 0.1% (0.0%, 0.2%) | 0.016 |
|  | India | 25 (0.1%) | 47 (0.2%) | - | 1.784 (0.989, 3.219) 0.1% (-0.0%, 0.2%) | 0.055 | NE | - |
|  | Tanzania | 51 (0.2%) | 94 (0.3%) | - | 1.719 (0.965, 3.062) 0.1% (-0.0%, 0.2%) | 0.066 | 1.466 (0.888, 2.422) 0.1% (-0.1%, 0.3%) | 0.135 |
|  |  | 51 (0.2%) | - | 124 (0.3%) | 2.089 (1.165, 3.745) 0.2% (0.0%, 0.3%) | 0.013 | 1.581 (0.951, 2.628) 0.1% (-0.0%, 0.3%) | 0.077 |
| Sensitivity analysis-referral caregiver and registry | Combined | - | - | - | - | - | - | - |
|  | India | - | - | - | - | - | - | - |
|  | Tanzania | 57 (0.2%) | 99 (0.3%) | - | 1.673 (0.940, 2.978) 0.1% (-0.0%, 0.3%) | 0.080 | 1.413 (0.855, 2.335) 0.1% (-0.1%, 0.3%) | 0.178 |
|  |  | 57 (0.2%) | - | 132 (0.4%) | 2.020 (1.117, 3.652) 0.2% (-0.0%, 0.3%) | 0.020 | 1.521 (0.904, 2.560) 0.1% (-0.1%, 0.3%) | 0.114 |
| Sensitivity analysis-referral only registry | Combined | 79 (0.1%) | 143 (0.3%) | - | 1.735 (1.132, 2.657) 0.1% (0.0%, 0.2%) | 0.011 | 1.635 (1.088, 2.456) 0.1% (0.0%, 0.2%) | 0.018 |
|  | India | 24 (0.1%) | 46 (0.2%) | - | 1.803 (0.981, 3.315) 0.1% (-0.0%, 0.2%) | 0.058 | NE | - |
|  | Tanzania | 55 (0.2%) | 97 (0.3%) | - | 1.690 (0.948, 3.013) 0.1% (-0.0%, 0.3%) | 0.075 | 1.438 (0.869, 2.381) 0.1% (-0.1%, 0.3%) | 0.158 |
|  |  | 55 (0.2%) | - | 131 (0.4%) | 2.085 (1.155, 3.764) 0.2% (0.0%, 0.3%) | 0.015 | 1.573 (0.944, 2.623) 0.1% (-0.0%, 0.3%) | 0.082 |
| Subgroup analysis - no severe diagnosis | Combined | 74 (0.1%) | 124 (0.2%) | - | 1.565 (1.020, 2.401) 0.1% (0.0%, 0.1%) | 0.040 | 1.467 (0.970, 2.219) 0.1% (-0.0%, 0.2%) | 0.069 |
| Subgroup analysis - severe diagnosis | Combined | 3 (0.3%) | 19 (1.4%) | - | 5.565 (1.511, 20.499) 1.9% (0.5%, 3.3%) | 0.010 | 4.040 (1.100, 14.836) 2.1% (-0.2%, 4.4%) | 0.035 |
| Appropriate referrals | | | | | | | | |
| Primary analysis ITT | Combined | 12 (0%) | 22 (0%) | - | 2.476 (0.862, 7.116) 0.0% (-0.0%, 0.1%) | 0.092 | NE | - |
|  | India | 0 (0%) | 6 (0%) | - | NE | - | NE | - |
|  | Tanzania | 12 (0%) | 16 (0%) | - | 1.647 (0.500, 5.430) 0.0% (-0.0%, 0.1%) | 0.412 | NE | - |
|  |  | 12 (0%) | - | 32 (0.1%) | 2.180 (0.782, 6.075) 0.0% (-0.0%, 0.1%) | 0.136 | NE | - |
| Primary analysis CC | Combined | 12 (0%) | 22 (0%) | - | 2.444 (0.854, 6.998) 0.0% (-0.0%, 0.1%) | 0.096 | NE | - |
|  | India | 0 (0%) | 6 (0%) | - | NE | - | NE | - |
|  | Tanzania | 12 (0%) | 16 (0%) | - | 1.628 (0.495, 5.351) 0.0% (-0.1%, 0.1%) | 0.422 | NE | - |
|  |  | 12 (0%) | - | 32 (0.1%) | 2.150 (0.772, 5.986) 0.0% (-0.0%, 0.1%) | 0.143 | NE | - |
| Sensitivity analysis-first encounters | Combined | 8 (0%) | 19 (0%) | - | 2.895 (0.924, 9.069) 0.0% (-0.0%, 0.1%) | 0.068 | NE | - |
|  | India | 0 (0%) | 6 (0%) | - | NE | - | NE | - |
|  | Tanzania | 8 (0%) | 13 (0%) | - | 2.035 (0.555, 7.463) 0.0% (-0.0%, 0.1%) | 0.284 | NE | - |
|  |  | 8 (0%) | - | 26 (0.1%) | 2.322 (0.702, 7.678) 0.0% (-0.0%, 0.1%) | 0.167 | NE | - |
| Sensitivity analysis-hosp cut-off Day0 | Combined | 10 (0%) | 19 (0%) | - | 2.817 (0.848, 9.364) 0.0% (-0.0%, 0.0%) | 0.091 | NE | - |
|  | India | 0 (0%) | 5 (0%) | - | NE | - | NE | - |
|  | Tanzania | 10 (0%) | 14 (0%) | - | 1.862 (0.502, 6.903) 0.0% (-0.0%, 0.1%) | 0.352 | NE | - |
|  |  | 10 (0%) | - | 27 (0.1%) | 2.280 (0.692, 7.508) 0.0% (-0.0%, 0.1%) | 0.175 | NE | - |
| Sensitivity analysis-hosp cut-off Day3 | Combined | 12 (0%) | 24 (0%) | - | 2.758 (0.961, 7.917) 0.0% (-0.0%, 0.1%) | 0.059 | NE | - |
|  | India | 0 (0%) | 7 (0%) | - | NE | - | NE | - |
|  | Tanzania | 12 (0%) | 17 (0.1%) | - | 1.805 (0.551, 5.917) 0.0% (-0.0%, 0.1%) | 0.329 | NE | - |
|  |  | 12 (0%) | - | 35 (0.1%) | 2.386 (0.860, 6.619) 0.0% (-0.0%, 0.1%) | 0.095 | NE | - |
| Sensitivity analysis-hosp cut-off Day7 | Combined | 13 (0%) | 24 (0%) | - | NE | - | NE | - |
|  | India | 0 (0%) | 7 (0%) | - | NE | - | NE | - |
|  | Tanzania | 13 (0%) | 17 (0.1%) | - | 1.714 (0.516, 5.693) 0.0% (-0.1%, 0.1%) | 0.379 | NE | - |
|  |  | 13 (0%) | - | 37 (0.1%) | 2.412 (0.871, 6.681) 0.1% (-0.0%, 0.1%) | 0.090 | NE | - |
| Sensitivity analysis-referral caregiver and registry | Combined | - | - | - | - | - | - | - |
|  | India | - | - | - | - | - | - | - |
|  | Tanzania | 7 (0%) | 12 (0%) | - | 2.096 (0.482, 9.112) 0.0% (-0.0%, 0.1%) | 0.324 | NE | - |
|  |  | 7 (0%) | - | 29 (0.1%) | 3.208 (0.819, 12.560) 0.0% (-0.0%, 0.1%) | 0.094 | NE | - |
| Sensitivity analysis-referral only registry | Combined | 10 (0%) | 22 (0%) | - | 2.738 (0.983, 7.629) 0.0% (-0.0%, 0.1%) | 0.054 | NE | - |
|  | India | 1 (0%) | 8 (0%) | - | NE | - | NE | - |
|  | Tanzania | 9 (0%) | 14 (0%) | - | 1.897 (0.506, 7.115) 0.0% (-0.0%, 0.1%) | 0.343 | NE | - |
|  |  | 9 (0%) | - | 30 (0.1%) | 2.543 (0.775, 8.342) 0.0% (-0.0%, 0.1%) | 0.124 | NE | - |
