# Supplementary material for: Effectiveness of introducing pulse oximetry and clinical decision support algorithms for the management of sick children in primary care in India and Tanzania on hospitalisation and mortality: the TIMCI pragmatic cluster randomised controlled trial
Source: eClinicalMedicine. 2025 Jul 3;85:103306. doi: 10.1016/j.eclinm.2025.103306 (PMC12271772; doi:10.1016/j.eclinm.2025.103306)
Supplement: 01_RCT_S5 [file mmc9.docx]

## Supplementary file S5 – Summary of secondary outcomes results

Similarly to primary outcomes, the secondary outcomes are assessed using generalised estimating equation for logistic regression, with facilities as clusters. Estimates for the intervention effect on the outcomes are shown in terms of odds ratios and risk difference (with the control arm being the reference category) and their associated 95% confidence intervals. Results from both univariate and multivariate models are presented when numbers allowed. Not estimable intervention effects are denoted in the summary table below as “NE”.

Multivariate models are adjusted for districts, facility type (dispensaries or PHCs/haelth centers or CHCs) and previous care or treatment at time of presenting at the facility (no/yes/unknown).

### Summary of secondary outcomes results - infants under 2 months of age

| Outcome | Analysis | N (%) Control | N (%) PO | N (%) PO+CDSA | Unadjusted | p-value | Adjusted | p-value |
| --- | --- | --- | --- | --- | --- | --- | --- | --- |
| Severe complications by Day28 | Combined | 22 (0.7%) | 46 (1.1%) | - | 1.684 (1.005, 2.821) 0.5% (0.0%, 0.9%) | 0.048 | NE | - |
|  | India | 8 (0.7%) | 15 (0.9%) | - | 2.372 (1.033, 5.447) 0.9% (0.1%, 1.7%) | 0.042 | NE | - |
|  | Tanzania | 8 (0.4%) | 12 (0.5%) | - | 1.231 (0.655, 2.314) 0.2% (-0.4%, 0.7%) | 0.518 | NE | - |
|  |  | 8 (0.4%) | - | 21 (0.9%) | 1.994 (1.075, 3.700) 0.7% (-0.0%, 1.4%) | 0.029 | NE | - |
| Urgent referrals | Combined | 28 (0.9%) | 62 (1.5%) | - | 2.232 (1.143, 4.357) 0.9% (0.0%, 1.8%) | 0.019 | 883 (0.979, 3.619) 0.8% (-0.1%, 1.6%) | 0.058 |
|  | India | 5 (0.4%) | 30 (1.9%) | - | 4.564 (1.513, 13.769) 1.6% (0.2%, 3.0%) | 0.007 | NE | - |
|  | Tanzania | 23 (1.1%) | 32 (1.3%) | - | 1.274 (0.537, 3.020) 0.3% (-1.0%, 1.5%) | 0.582 | NE | - |
|  |  | 23 (1.1%) | - | 57 (2.4%) | 2.508 (1.152, 5.460) 1.5% (-0.4%, 3.4%) | 0.021 | NE | - |
| Completed referrals | Combined | 3 (0.1%) | 18 (0.4%) | - | 5.680 (1.358, 23.754) 0.4% (0.1%, 0.7%) | 0.017 | NE | - |
|  | India | 1 (0.1%) | 10 (0.6%) | - | 6.476 (0.765, 54.795) 0.5% (-0.0%, 1.1%) | 0.086 | NE | - |
|  | Tanzania | 2 (0.1%) | 8 (0.3%) | - | 4.105 (0.620, 27.159) 0.3% (-0.1%, 0.6%) | 0.143 | NE | - |
|  |  | 2 (0.1%) | - | 9 (0.4%) | 4.781 (0.715, 31.965) 0.3% (-0.1%, 0.7%) | 0.106 | NE | - |
| Referrals with non-severe disease | Combined | 19 (0.6%) | 39 (1%) | - | 2.000 (0.952, 4.201) 0.6% (-0.1%, 1.3%) | 0.067 | NE | - |
|  | India | 4 (0.3%) | 21 (1.3%) | - | 3.386 (1.105, 10.369) 1.1% (-0.1%, 2.2%) | 0.033 | NE | - |
|  | Tanzania | 15 (0.7%) | 18 (0.7%) | - | 1.144 (0.403, 3.250) 0.1% (-0.8%, 1.0%) | 0.800 | NE | - |
|  |  | 15 (0.7%) | - | 37 (1.6%) | 2.365 (0.902, 6.200) 0.9% (-0.6%, 2.4%) | 0.080 | NE | - |
| Cure rate by Day7 | Combined | 2177 (68.3%) | 2773 (69.1%) | - | 0.924 (0.698, 1.224) -1.6% (-7.1%, 4.0%) | 0.581 | 0.945 (0.724, 1.235) -1.1% (-6.4%, 4.2%) | 0.681 |
|  | India | 887 (75.4%) | 1245 (78.7%) | - | 0.897 (0.607, 1.324) -1.9% (-8.9%, 5.0%) | 0.584 | 0.892 (0.609, 1.307) -1.8% (-7.9%, 4.3%) | 0.558 |
|  | Tanzania | 1290 (64.1%) | 1528 (62.9%) | - | 0.990 (0.680, 1.441) -0.2% (-10.1%, 9.6%) | 0.957 | 1.099 (0.756, 1.597) 2.1% (-7.8%, 11.9%) | 0.621 |
|  |  | 1290 (64.1%) | - | 1351 (56.6%) | 0.704 (0.449, 1.106) -8.1% (-20.7%, 4.5%) | 0.128 | 0.733 (0.482, 1.114) -7.2% (-18.9%, 4.4%) | 0.145 |
| Antibiotics prescription | Combined | 1780 (55.8%) | 2033 (50.7%) | - | 0.931 (0.700, 1.239) -1.8% (-8.9%, 5.3%) | 0.625 | 0.977 (0.742, 1.287) -0.6% (-7.2%, 6.1%) | 0.871 |
|  | India | 609 (51.8%) | 655 (41.4%) | - | 1.038 (0.705, 1.528) 0.9% (-8.7%, 10.6%) | 0.851 | 1.044 (0.714, 1.527) 1.0% (-8.0%, 10.0%) | 0.824 |
|  | Tanzania | 1171 (58.2%) | 1378 (56.7%) | - | 0.892 (0.598, 1.332) -2.8% (-14.5%, 8.9%) | 0.577 | 0.996 (0.675, 1.469) -0.1% (-11.2%, 11.1%) | 0.984 |
|  |  | 1171 (58.2%) | - | 1399 (58.6%) | 1.037 (0.703, 1.529) 0.9% (-10.4%, 12.2%) | 0.856 | 1.096 (0.743, 1.619) 2.2% (-8.9%, 13.3%) | 0.643 |

### Summary of secondary outcomes results - children 2-59 months of age

| Outcome | Analysis | N (%) Control | N (%) PO | N (%) PO+CDSA | Unadjusted | p-value | Adjusted | p-value |
| --- | --- | --- | --- | --- | --- | --- | --- | --- |
| Severe complications by Day28 | Combined | 136 (0.3%) | 221 (0.4%) | - | 1.546 (1.049, 2.278) 0.1% (0.0%, 0.2%) | 0.028 | 1.517 (1.047, 2.198) 0.1% (0.0%, 0.3%) | 0.028 |
|  | India | 37 (0.2%) | 68 (0.3%) | - | 1.704 (0.999, 2.906) 0.1% (-0.0%, 0.2%) | 0.050 | NE | - |
|  | Tanzania | 99 (0.3%) | 153 (0.5%) | - | 1.459 (0.871, 2.441) 0.1% (-0.1%, 0.3%) | 0.151 | 1.251 (0.771, 2.030) 0.1% (-0.2%, 0.4%) | 0.365 |
|  |  | 99 (0.3%) | - | 205 (0.6%) | 1.860 (1.085, 3.187) 0.2% (-0.0%, 0.5%) | 0.024 | 1.429 (0.888, 2.298) 0.2% (-0.1%, 0.4%) | 0.141 |
| Urgent referrals | Combined | 126 (0.2%) | 344 (0.6%) | - | 2.499 (1.235, 5.057) 0.2% (0.0%, 0.4%) | 0.011 | NE | - |
|  | India | 14 (0.1%) | 65 (0.3%) | - | 3.771 (1.577, 9.017) 0.2% (0.0%, 0.3%) | 0.003 | NE | - |
|  | Tanzania | 112 (0.4%) | 279 (0.8%) | - | 2.067 (0.803, 5.322) 0.3% (-0.3%, 0.9%) | 0.133 | NE | - |
|  |  | 112 (0.4%) | - | 645 (1.8%) | 4.931 (1.706, 14.252) 1.2% (-0.4%, 2.8%) | 0.003 | NE | - |
| Completed referrals by Day7 | Combined | 17 (0%) | 41 (0.1%) | - | 3.268 (1.441, 7.412) 0.1% (0.0%, 0.1%) | 0.005 | NE | - |
|  | India | 2 (0%) | 17 (0.1%) | - | NE | - | NE | - |
|  | Tanzania | 15 (0%) | 24 (0.1%) | - | 2.134 (0.704, 6.468) 0.0% (-0.1%, 0.1%) | 0.180 | NE | - |
|  |  | 15 (0%) | - | 48 (0.1%) | 2.711 (1.048, 7.015) 0.1% (-0.0%, 0.2%) | 0.040 | NE | - |
| Referrals with non-severe disease | Combined | 74 (0.1%) | 161 (0.3%) | - | 1.858 (0.907, 3.807) 0.1% (-0.0%, 0.2%) | 0.090 | NE | - |
|  | India | 13 (0.1%) | 40 (0.2%) | - | 2.463 (0.956, 6.347) 0.1% (-0.0%, 0.2%) | 0.062 | NE | - |
|  | Tanzania | 61 (0.2%) | 121 (0.4%) | - | 1.513 (0.538, 4.253) 0.1% (-0.2%, 0.4%) | 0.432 | NE | - |
|  |  | 61 (0.2%) | - | 410 (1.1%) | 5.638 (1.381, 23.013) 0.8% (-0.6%, 2.1%) | 0.016 | NE | - |
| Cure rate by Day7 | Combined | 38825 (71.5%) | 41969 (73.7%) | - | 1.116 (0.895, 1.392) 2.0% (-2.0%, 6.1%) | 0.329 | 1.116 (0.906, 1.374) 2.0% (-1.8%, 5.9%) | 0.303 |
|  | India | 17941 (78.4%) | 18624 (79.6%) | - | 1.100 (0.840, 1.441) 1.6% (-3.0%, 6.3%) | 0.487 | 1.063 (0.818, 1.381) 1.1% (-3.6%, 5.9%) | 0.649 |
|  | Tanzania | 20884 (66.4%) | 23345 (69.5%) | - | 1.142 (0.815, 1.600) 2.8% (-5.6%, 11.2%) | 0.440 | 1.257 (0.908, 1.740) 4.8% (-3.4%, 12.9%) | 0.169 |
|  |  | 20884 (66.4%) | - | 22356 (60.7%) | 0.750 (0.486, 1.158) -6.4% (-18.3%, 5.4%) | 0.195 | 0.761 (0.506, 1.143) -6.2% (-17.2%, 4.8%) | 0.188 |
| Antibiotics prescription | Combined | 40291 (74.2%) | 40695 (71.4%) | - | 0.817 (0.621, 1.076) -4.1% (-9.6%, 1.5%) | 0.150 | 0.802 (0.611, 1.054) -4.4% (-9.9%, 1.0%) | 0.113 |
|  | India | 17233 (75.3%) | 16430 (70.3%) | - | 0.823 (0.576, 1.175) -4.0% (-11.3%, 3.3%) | 0.284 | 0.799 (0.561, 1.140) -4.5% (-11.6%, 2.7%) | 0.216 |
|  | Tanzania | 23058 (73.4%) | 24265 (72.3%) | - | 0.809 (0.546, 1.198) -4.2% (-13.5%, 5.0%) | 0.290 | 0.833 (0.566, 1.227) -3.6% (-12.4%, 5.3%) | 0.354 |
|  |  | 23058 (73.4%) | - | 26472 (71.9%) | 0.869 (0.597, 1.265) -2.8% (-11.4%, 5.9%) | 0.463 | 0.862 (0.560, 1.328) -2.9% (-12.7%, 7.0%) | 0.501 |
| Febrile children tested for malaria | Combined | 15903 (42%) | 17650 (43.4%) | - | 1.048 (0.545, 2.015) 0.8% (-9.8%, 11.3%) | 0.889 | 1.370 (0.680, 2.760) 3.2% (-3.8%, 10.1%) | 0.378 |
|  | India | 191 (1.3%) | 140 (0.9%) | - | 0.619 (0.165, 2.320) -0.4% (-1.7%, 0.9%) | 0.477 | NE | - |
|  | Tanzania | 15712 (67.4%) | 17510 (70.8%) | - | 1.188 (0.668, 2.114) 3.7% (-10.9%, 18.3%) | 0.558 | 1.103 (0.569, 2.138) 2.1% (-14.5%, 18.6%) | 0.772 |
|  |  | 15712 (67.4%) | - | 19566 (70.3%) | 1.043 (0.614, 1.771) 0.9% (-12.9%, 14.7%) | 0.877 | 1.502 (0.787, 2.868) 8.0% (-7.3%, 23.3%) | 0.218 |
| Antimalarial prescription for children tested positive | Combined | 4785 (95.8%) | 3572 (90.1%) | - | 0.469 (0.200, 1.099) -11.0% (-24.1%, 2.1%) | 0.081 | NE | - |
|  | India | - | - | - | - | - | - | - |
|  | Tanzania | 4785 (95.8%) | 3572 (90.2%) | - | 0.509 (0.208, 1.248) -7.7% (-21.1%, 5.7%) | 0.140 | 0.419 (0.133, 1.325) -9.5% (-25.2%, 6.2%) | 0.139 |
|  |  | 4785 (95.8%) | - | 3955 (88.2%) | 0.674 (0.272, 1.670) -4.0% (-15.7%, 7.7%) | 0.394 | 0.674 (0.272, 1.670) -4.0% (-15.7%, 7.7%) | 0.394 |
| Antimalarial prescription for children tested negative | Combined | 163 (1.3%) | 201 (1.3%) | - | 1.231 (0.491, 3.085) 0.3% (-1.2%, 1.8%) | 0.658 | NE | - |
|  | India | - | - | - | - | - | - | - |
|  | Tanzania | 163 (1.3%) | 200 (1.3%) | - | 1.183 (0.459, 3.048) 0.3% (-1.7%, 2.2%) | 0.728 | 1.504 (0.626, 3.615) 0.7% (-1.3%, 2.6%) | 0.361 |
|  |  | 163 (1.3%) | - | 418 (2.4%) | 1.576 (0.871, 2.852) 0.8% (-0.6%, 2.3%) | 0.132 | 1.820 (1.130, 2.929) 1.1% (-0.2%, 2.3%) | 0.014 |
| Antimalarial prescription for children untested | Combined | 172 (0.5%) | 162 (0.4%) | - | 0.804 (0.204, 3.170) -0.1% (-1.0%, 0.7%) | 0.756 | NE | - |
|  | India | - | - | - | - | - | - | - |
|  | Tanzania | 170 (1.2%) | 160 (1.1%) | - | 0.626 (0.141, 2.792) -1.1% (-5.6%, 3.4%) | 0.540 | 1.978 (0.549, 7.128) 1.2% (-1.6%, 4.0%) | 0.297 |
|  |  | 170 (1.2%) | - | 357 (2.4%) | 1.249 (0.323, 4.829) 0.7% (-4.4%, 5.8%) | 0.747 | 2.895 (0.891, 9.406) 2.3% (-0.9%, 5.4%) | 0.077 |
