# Supplementary material for: Effectiveness of introducing pulse oximetry and clinical decision support algorithms for the management of sick children in primary care in India and Tanzania on hospitalisation and mortality: the TIMCI pragmatic cluster randomised controlled trial
Source: eClinicalMedicine. 2025 Jul 3;85:103306. doi: 10.1016/j.eclinm.2025.103306 (PMC12271772; doi:10.1016/j.eclinm.2025.103306)
Supplement: 01_RCT_S6 [file mmc10.docx]

Supplementary file S6 – Follow-up rates and hospital records availability for hypoxaemia-related outcomes

|  | **1-59 days** | | | | **2-59 months** | | | |
| --- | --- | --- | --- | --- | --- | --- | --- | --- |
|  | **PO** | | | **PO+CDSA** | **PO** | | | **PO+CDSA** |
| Characteristics | Cross-country (n = 4 012) | India (n = 1 582) | Tanzania (n = 2 430) | Tanzania (n=2 386) | Cross-country (n = 56 968) | India (n = 23 384) | Tanzania (n = 33 584) | Tanzania (n = 36 805) |
| **Day 7 and hospital follow-up rates in referred severely hypoxaemic children, % (n)** | | | | | | | | |
| Day 7 follow-up among referred | 9/11 (81·8%) | 4/6 (66·7%) | 5/5 (100·0%) | 6/8 (75·0%) | 28/30 (93·3%) | 14/14 (100·0%) | 14/16 (87·5%) | 40/46 (87·0%) |
| Hospital record found among referred | 3/11 (27·3%) | 0/6 (0·0%) | 3/5 (60·0%) | 1/8 (12·5%) | 7/30 (23·3%) | 4/14(28·6%) | 3/16 (18·8%) | 9/46 (19·6%) |
| Hospital record found among those who attended a higher level of care | 3/6 (50·0%) | 0/3 (0·0%) | 3/3 (100·0%) |  | 7/9 (77·8%) | 4/6 (66·7%) | 3/3 (100·0%) | 7/9  (77·8%) |
